# Joint association of sedentary behavior and vitamin D status with mortality among cancer survivors

**DOI:** 10.1186/s12916-023-03118-9

**Published:** 2023-10-31

**Authors:** Yu Yu, Sijing Cheng, Hao Huang, Yu Deng, Chi Cai, Min Gu, Xuhua Chen, Hongxia Niu, Wei Hua

**Affiliations:** Cardiac Arrhythmia Center, Department of Cardiology, National Center for Cardiovascular Diseases, State Key Laboratory of Cardiovascular Disease, Fuwai Hospital, Chinese Academy of Medical Sciences and Peking Union Medical College, No. 167 Bei Li Shi Rd, Xicheng District, Beijing, 100037 China

**Keywords:** Joint association, Cancer, Sedentary behavior, Vitamin D, Survival

## Abstract

**Background:**

Sedentary behavior and vitamin D deficiency are independent risk factors for mortality in cancer survivors, but their joint association with mortality has not been investigated.

**Methods:**

We analyzed data from 2914 cancer survivors who participated in the National Health and Nutrition Examination Survey (2007–2018) and followed up with them until December 31, 2019. Sedentary behavior was assessed by self-reported daily hours of sitting, and vitamin D status was measured by serum total 25-hydroxyvitamin D (25(OH)D) levels.

**Results:**

Among 2914 cancer survivors, vitamin D deficiency was more prevalent in those with prolonged daily sitting time. During up to 13.2 years (median, 5.6 years) of follow-up, there were 676 deaths (cancer, 226; cardiovascular disease, 142; other causes, 308). The prolonged sitting time was associated with a higher risk of all-cause and noncancer mortality, and vitamin D deficiency was associated with a higher risk of all-cause and cancer mortality. Furthermore, cancer survivors with both prolonged sitting time (≥ 6 h/day) and vitamin D deficiency had a significantly higher risk of all-cause (HR, 2.05; 95% CI: 1.54–2.72), cancer (HR, 2.33; 95% CI, 1.47–3.70), and noncancer mortality (HR, 1.91; 95% CI, 1.33–2.74) than those with neither risk factor after adjustment for potential confounders.

**Conclusions:**

In a nationally representative sample of U.S. cancer survivors, the joint presence of sedentary behavior and vitamin D deficiency was significantly associated with an increased risk of all-cause and cancer-specific mortality.

**Supplementary Information:**

The online version contains supplementary material available at 10.1186/s12916-023-03118-9.

## Background

The number of cancer survivors worldwide has been rapidly increasing in recent years, with 19.3 million new cases in 2020 and a projected increase to 28.4 million new cases by 2040 [1]. While modern medicine has greatly increased the lifespan of cancer survivors, there remains a persistent challenge in improving their long-term outcomes [2]. Therefore, there is a pressing need to develop effective therapeutic strategies to enhance the survival of cancer survivors [3, 4]. Research indicates that sedentary behavior is highly prevalent among cancer survivors. According to a study by Phillips et al. [5], it was reported that more than 60% of adult cancer survivors in the USA engage in more than 8 h of sedentary activity per day, which significantly exceeds the daily average of the general population. Similarly, Lynch et al. [6] reported that the average daily sitting time was 9.3 h in cancer survivors, with 66% of them exceeding this average. These findings underscore the high prevalence of sedentary behavior among cancer survivors and highlight sedentary behavior is a significant concern in this population. The high prevalence of prolonged sitting time among cancer patients could be attributed to the general weakness and fatigue induced by tumors and oncological treatments, prompting these individuals to sit more frequently to mitigate their discomfort [7, 8]. Extant literature indicates that sustained sedentary behavior is correlated with an increased risk of cancer and exerts a deleterious impact on the survival outcomes of cancer survivors [4, 9]. Consequently, determining an optimal duration for sedentary activity could offer significant survival advantages to this population.

Vitamin D is an essential fat-soluble vitamin that plays a crucial role in regulating blood calcium and phosphorus, bone metabolism, and immune balance [10, 11]. As research has progressed, increasing evidence shows that vitamin D deficiency is closely related to a range of conditions, including CVD, diabetes, respiratory disease, multiple sclerosis, periodontal disease, COVID-19, and various types of cancer [12–14]. Additionally, circulating vitamin D levels are significantly associated with the survival outcomes in cancer survivors, although existing studies have yielded inconsistent conclusions. Several studies have reported a positive association between vitamin D deficiency and mortality risk in patients with breast, lung, stomach, and prostate cancer [15–18]. However, a randomized controlled trial by Manson et al. [12] did not find that higher vitamin D levels improved the survival prognosis of cancer survivors. These inconsistent studies make it necessary to re-investigate the relationship between vitamin D levels and mortality risk in cancer survivors.

Despite the growing interest in these individual factors, a glaring research gap emerges: the lack of studies exploring the combined effects of sedentary behavior and vitamin D status on cancer survivors’ mortality. This gap becomes even more pertinent considering the potential for sedentary behavior to decrease vitamin D synthesis and bioavailability due to reduced sunlight exposure and obesity [19–21]. Furthermore, sedentary behavior and vitamin D deficiency often co-occur, especially among cancer survivors who may experience physical limitations and fatigue [22]. Considering the possible interplay between sedentary behavior and vitamin D levels, a combined evaluation of their effects could offer a nuanced understanding of their influence on survival rates among cancer survivors. Crucially, the joint analysis might pinpoint specific subgroups of survivors, particularly those exhibiting both pronounced sedentary habits and deficient vitamin D levels, who face a heightened risk.

In view of the extant research disparities and identified gaps in the literature, our study investigates the prevalence of prolonged sitting time and vitamin D deficiency in a nationally representative U.S. sample of cancer survivors. Specifically, we examine their independent and joint associations with all-cause, cancer-related, and non-cancer-related mortality.

## Methods

### Study population

**Fig. 1:**
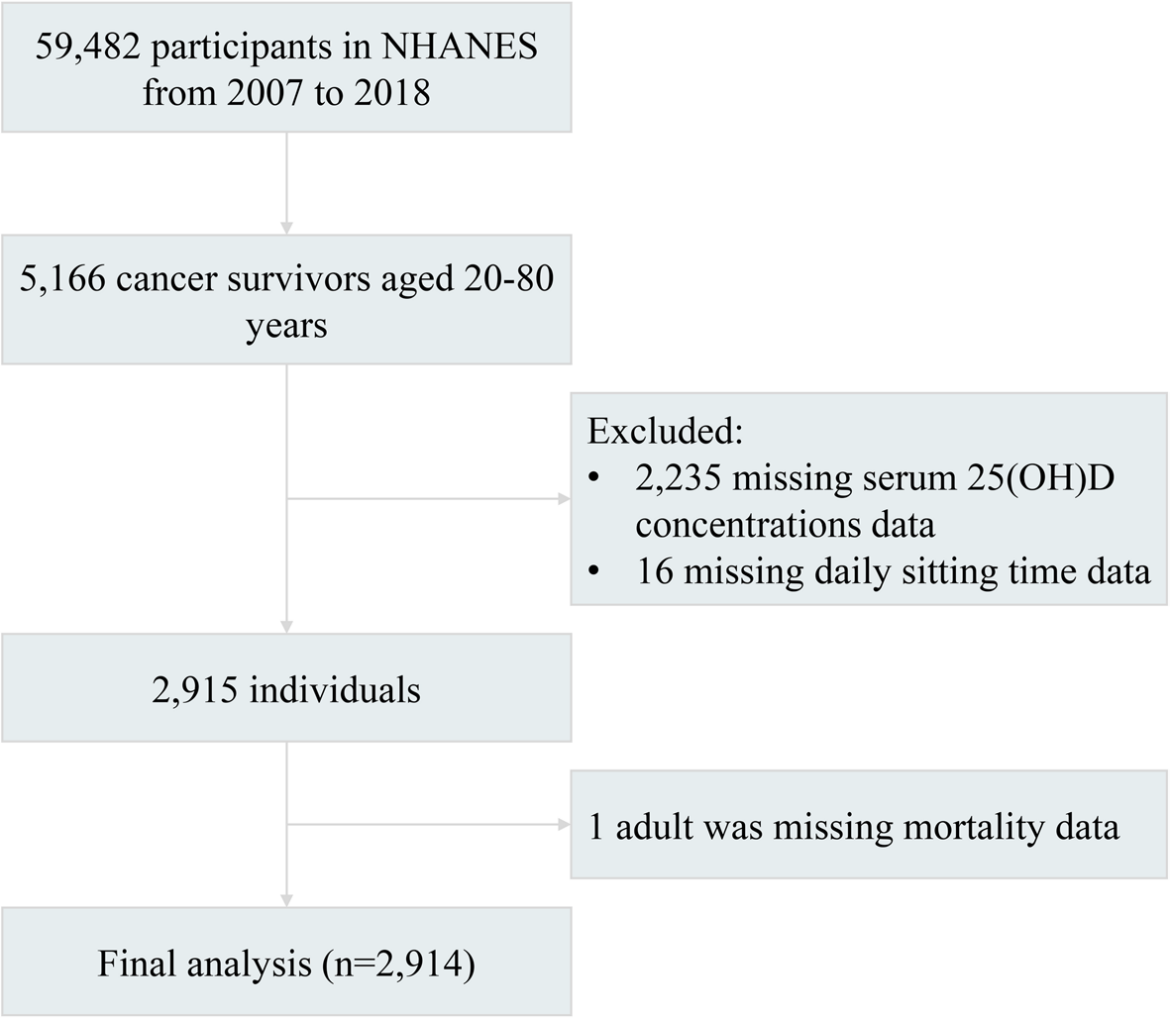
Flow chart of study participants

This study used data collected from the National Health and Nutrition Examination Survey (NHANES), a program managed by the Centers for Disease Control and Prevention (CDC) and the National Centers for Health Statistics (NCHS) in the USA. In brief, the program aims to assess the health and nutritional status of the U.S. population and follows the STROBE guidelines for reporting observational studies. The NHANES study protocol was approved by the NCHS Research Ethics Review Board, and written informed consent was provided by all participants. We extracted survey data from the NHANES website covering six survey cycles between 2007 and 2018 (https://www.cdc.gov/nchs/nhanes/index.htm). The data analyzed in this study included demographic information, health status, examination data, laboratory data, and questionnaire data. A total of 5166 self-reported cancer survivors were included in the NHANES cohort between 2007 and 2018. After excluding those with missing data, we included 2914 eligible cancer survivors in the final analysis (Fig. 1).

### Diagnosis of cancer

Cancer survivors were identified through the NHANES questionnaire: “Has a doctor or other health professional ever told you that you have cancer, and if yes, what type of cancer did you have?” and “How old were you when this cancer was first diagnosed?” Cancer types in our study were further classified into nine categories of cancer: gynecologic tumors (breast, cervical, ovarian, uterine), urologic tumors in males (prostate, testicular), head and neck tumors (laryngeal/tracheal, oral/tongue/lip, thyroid), respiratory system tumors (lung, laryngeal/tracheal), gastrointestinal tumors (colorectal, esophageal, gallbladder, hepatocellular, pancreatic, rectal, gastric), urologic tumors (bladder, renal cell), skin cancers (melanoma, non-melanoma), hematologic malignancies (leukemia, lymphoma, other blood cancers), and other cancers. The reliability and accuracy of self-reported cancer diagnoses in NHANES have been assessed in prior studies, suggesting that the agreement between self-reported data and medical records is generally good for most common cancer types [23, 24].

### Daily sitting time, physical activity, and vitamin D measurements

In the NHANES study, physical activity levels and sedentary duration were evaluated using a self-administered physical activity questionnaire (PAQ). The PAQ utilized in NHANES has been validated and demonstrated moderate to good test–retest reliability [25–27]. Within this questionnaire, sitting time was ascertained by asking participants about their time spent sitting at home, at school, at work, commuting, or with friends throughout a typical day. In accordance with recent literature, the daily sitting time for cancer survivors in this study was categorized into predefined thresholds: less than 4 h, 4–6 h, 6–8 h, and more than 8 h [4, 28, 29]. The self-reported physical activity (PA) in NHANES was gathered using a questionnaire that asked about the frequency, duration, and intensity of physical activities. The amount of PA in our study was defined as the time of moderate-intensity PA plus twice the time of vigorous-intensity PA in the past 30 days. According the 2018 Physical Activity Guidelines for Americans, if the PA was more than 150 min per week, it was classified as active, otherwise, as inactive [30].

Vitamin D levels were determined from a single baseline serum sample collected from NHANES participants. The serum samples were processed, stored at –30 °C, and shipped to the Nutritional Biomarkers Branch of the Division of Laboratory Sciences at the National Center for Environmental Health. Researchers measured vitamin D concentration using total serum 25-hydroxyvitamin D [25(OH)D] levels, which is the sum of the 25(OH)D2 and 25(OH)D3 components. The total 25(OH)D levels were measured by high-performance liquid chromatography-tandem mass spectrometry (HPLC–MS/MS) using the CDC method. Detailed information about the 25(OH)D concentration measurement method is available on the NHANES website.

### Ascertainment of mortality

The death data used in this study were recorded from the National Death Index (NDI) death certificate records provided by NCHS, and the linked mortality files were updated to December 31, 2019. The study outcomes were all-cause mortality and cause-specific mortality due to cancer and noncancer causes. The causes of death were identified based on the International Classification of Diseases, 10th revision (ICD-10). All-cause mortality was defined as death from any cause, which encompasses cancer (C00-C97), cardiovascular disease (I00-I09, I11, I13, I20-I51), cerebrovascular disease (I60-I69), respiratory disease (J10-J18, J40-J47), and other causes. During follow-up, death due to malignancy was defined as cancer mortality (C00-C97). Deaths from any form of malignancy were classified as cancer mortality (C00-C97). The follow-up period was calculated from the baseline interview to the date of death or December 31, 2019.

### Assessment of covariates

The covariates included in this study comprised demographic information, health behavior, physical examination, and medical history. Demographic information was collected through self-report on NHANES questionnaires, which consisted of basic information, such as age, sex, race/ethnicity (non-Hispanic White, Hispanic, non-Hispanic Black, other), marital status, and education level. The ratio of family income to the poverty threshold based on family size and composition was used to estimate the family poverty income ratio. Body mass index was calculated by dividing weight (in kilograms) by the square of height (in meters) and classified as < 25, 25.0–29.9, or ≥ 30 kg/m^2^ [31]. For smoking status, participants were categorized as never smokers, former smokers, and current smokers. Former smokers were further divided based on pack-years: less than 10 pack-years and more than 10 pack-years. Similarly, current smokers were subdivided based on their daily cigarette intake: less than 10 cigarettes/day and over 10 cigarettes/day (Additional file 1: Table S1). Hypertension was determined if their blood pressure (BP) was measured to be over 140/90 mm Hg or if they were taking antihypertensive medication. Diabetes was diagnosed based on laboratory measurements of fasting plasma glucose and hemoglobin A1c, self-reported medication use, or a previous diagnosis of diabetes by a healthcare provider. Coronary heart disease was assessed through self-reported medical history and physical examination.

### Statistical analysis

This study’s statistical analyses were conducted in accordance with CDC guidelines (https://wwwn.cdc.gov/nchs/nhanes/tutorials/default.aspx). Since NHANES employs a complex multistage stratified probability survey design, the statistical analysis incorporated sample weights, clustering, and stratification. The baseline characteristics were displayed according to sitting time classification (< 4, 4 to < 6, 6 to 8, > 8 h/day), with the survey-weighted mean (95% confidence interval [CI]) to report continuous variables and percentages with their 95% CI to present categorical variables. Given the biological interrelationship and independent associations of both sedentary behavior and vitamin D status with cancer outcomes, this study mainly investigates their joint association with mortality among cancer survivors. In addition to the primary Cox proportional hazards models, interaction terms were also introduced to evaluate whether the combined effects of sedentary behavior and vitamin D status exceeded the cumulative effects of the two in the additive and multiplicative scales. Specifically, for the additive scale interaction, we computed the relative excess risk due to interaction (RERI) following the guidelines proposed by Knol and VanderWeele [32]. For the multiplicative scale, we used the ratio of odds ratios (ROR) to evaluate the interaction on the multiplicative scale. Confounding variables were selected based on three criteria: clinical relevance, a *P*-value less than 0.05 in univariate analysis, and the availability of sufficient event data to construct a robust regression model. Clinical relevance was established by employing a multifaceted approach that included directed acyclic graphs (DAGs), comprehensive literature reviews, and consultations with subject-matter experts [33] (Additional file 1: Fig. S1). In sensitivity analyses, we excluded cancer survivors with less than 3 years of follow-up to test the robustness of the results. Additionally, we conducted subgroup analyses to examine the stability of the results across age (< 65 years, ≥ 65 years), sex (male, female), cancer types, PA (active, inactive), baseline years (2007–2012, 2013–2018), and smoking status (never smoked; former smoker, light; former smoker; current smoker, light; current smoker, heavy).

All analyses were performed using the statistical packages R version 4.0.2 and SPSS (IBM) version 23, and a two-tailed *P* < 0.05 was regarded as statistically significant.

## Results

### Baseline characteristics

**Fig. 2:**
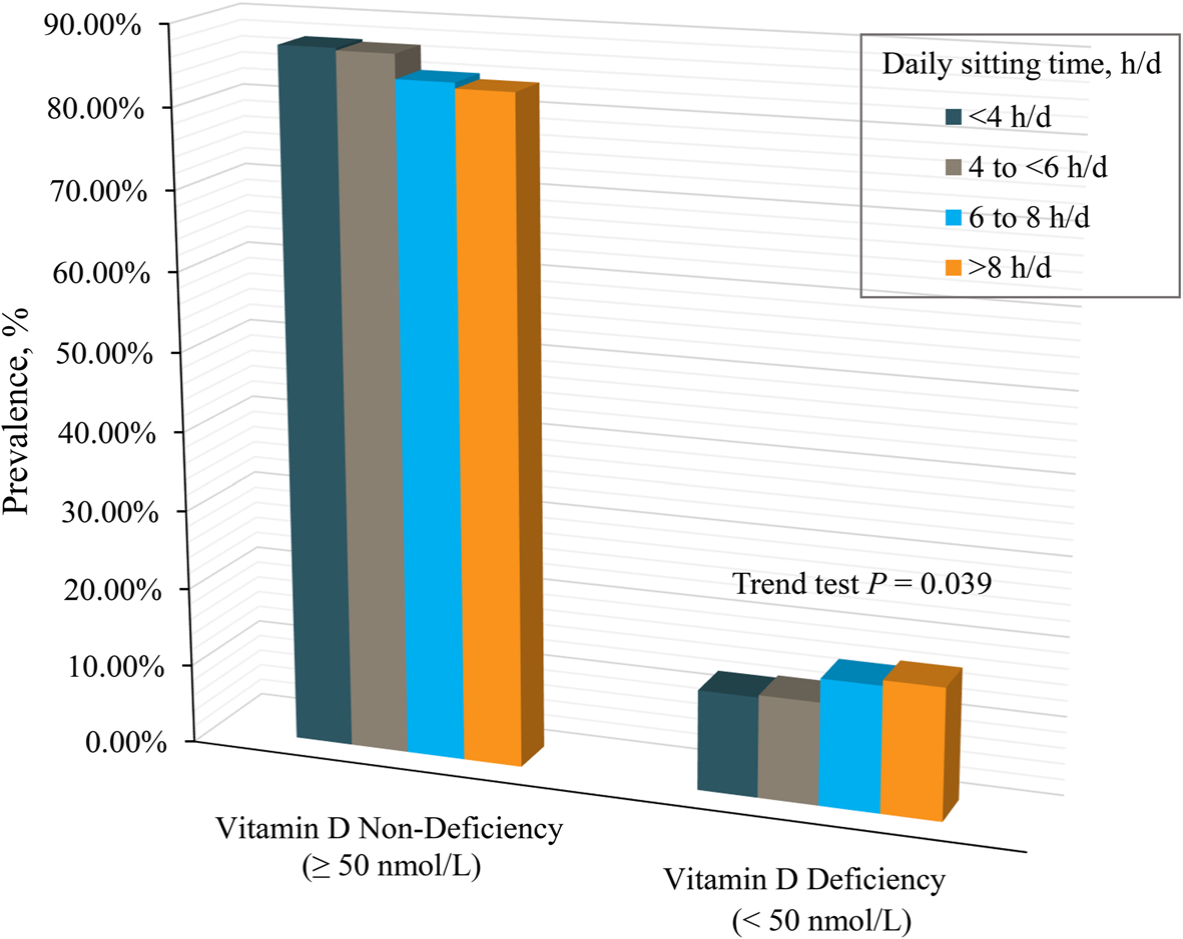
Joint prevalence of daily sitting time and vitamin D status in a nationally representative sample of US cancer survivors, NHANES 2007 to 2018

A total of 2914 cancer survivors were included in the study cohort (weighted population, 21,542,524; weighted mean age [SE] 62.7 [0.4] years; weighted male proportion 43.8%). Among these participants, 1953 (85.9%) were non-Hispanic White, 396 (5.3%) were Hispanic, 402 (4.9%) were non-Hispanic Black, and 163 (3.9%) were of other races. The mean daily sitting time was 6.6 [0.1] h per day, and the mean concentration of vitamin D was 81.6 [0.9] nmol/L. Participants with higher daily sitting time (> 8 h per day) were more likely to be male, non-Hispanic White, never married, more educated, have a higher family poverty income ratio, be obese, inactive, and have hypertension, diabetes, and vitamin D deficiency (Table 1). Importantly, there was a significant increasing trend in the prevalence of vitamin D deficiency among cancer survivors as daily sitting time increased (Trend test *P* = 0.039) (Fig. 2).

**Table 1:** Baseline characteristics of US cancer survivors and stratified by groups of daily sitting time, NHANES 2007 to 2018

Characteristic	No. of participants by daily sitting time (weighted %)^a^				
	**All**	** < 4 h/d**	**4 to < 6 h/d**	**6 to 8 h/d**	** > 8 h/d**
Participant	2914	980	733	570	631
Age, year	(2914) 62.7	(980) 60.4	(733) 65.7	(570) 64.2	(631) 61.5
Sex					
Men	(1381) 43.8	(446) 41.2	(343) 41.4	(289) 48.6	(303) 45.5
Women	(1533) 56.2	(534) 58.8	(390) 58.6	(281) 51.4	(328) 54.5
Race and ethnicity					
Non-Hispanic White	(1953) 85.9	(566) 81.6	(518) 86.9	(395) 86.5	(474) 89.7
Hispanic	(396) 5.3	(219) 8.9	(82) 5.0	(56) 3.8	(39) 2.5
Non-Hispanic Black	(402) 4.9	(138) 5.1	(93) 4.6	(90) 6.1	(81) 4.3
Other^b^	(163) 3.9	(57) 4.4	(40) 3.5	(29) 3.7	(37) 3.6
Baseline year^c^					
2007–2012	(1431) 43.5	(534) 47.9	(371) 43.5	(260) 41.0	(266) 40.1
2013–2018	(1483) 56.5	(446) 52.1	(362) 56.5	(310) 59.0	(365) 59.9
Marital status					
Married	(1731) 65.3	(606) 66.6	(445) 67.3	(322) 64.1	(358) 62.7
Widowed	(503) 13.8	(144) 11.6	(137) 16.5	(104) 14.6	(118) 13.1
Divorced or separated	(479) 14.7	(165) 16.0	(101) 10.9	(103) 15.5	(110) 16.3
Never married	(198) 6.2	(64) 5.8	(50) 5.4	(40) 5.8	(44) 7.9
Education attainment					
< High school	(253) 3.9	(115) 5.7	(53) 3.2	(48) 3.2	(37) 2.8
High school	(992) 28.6	(368) 33.6	(259) 31.3	(196) 28.5	(169) 19.9
> High school	(1667) 67.5	(497) 60.7	(420) 65.5	(326) 68.2	(424) 77.3
Family poverty income ratio	(2669) 3.3	(876) 3.2	(686) 3.2	(528) 3.3	(579) 3.6
BMI, kg/m^2^					
< 25	(769) 27.9	(277) 32.3	(209) 30.3	(141) 24.7	(142) 22.5
25 to < 30	(994) 34.5	(362) 37.2	(260) 35.4	(190) 32.4	(182) 32.0
≥ 30	(1099) 37.5	(325) 30.5	(257) 34.3	(233) 42.8	(284) 45.6
LTPA, min/wk					
≥ 150 (active)	(834) 35.0	(311) 38.5	(224) 36.0	(155) 34.5	(144) 29.9
< 150 (inactive)	(2080) 65.0	(669) 61.5	(509) 64.0	(415) 65.5	(487) 70.1
Smoking					
Never	(1333) 47.0	(459) 43.8	(323) 46.8	(271) 49.6	(280) 49.2
Former	(1115) 37.3	(353) 38.2	(285) 35.7	(218) 36.7	(259) 38.2
Current	(464) 15.7	(168) 18.0	(123) 17.5	(81) 13.7	(92) 12.6
Alcohol use					
Never or former	(892) 27.1	(313) 27.8	(217) 27.4	(193) 29.9	(169) 23.6
Mild to moderate	(1449) 62.5	(466) 60.4	(374) 63.1	(275) 61.3	(334) 65.5
Heavy	(241) 10.4	(93) 11.8	(54) 9.4	(45) 8.8	(49) 10.9
Hypertension	(1896) 58.6	(606) 55.4	(489) 60.4	(392) 61.6	(409) 58.3
Diabetes	(840) 23.6	(263) 20.8	(207) 21.9	(175) 26.5	(195) 26.5
CHD	(283) 8.1	(75) 6.5	(73) 7.7	(70) 12.2	(65) 7.3
Vitamin D, nmol/L					
≥ 50 (non-deficiency)	(2348) 85.6	(803) 87.3	(598) 87.0	(452) 84.0	(495) 83.3
< 50 (deficiency)	(566) 14.4	(177) 12.7	(135) 13.0	(118) 16.0	(136) 16.7

*BMI* body mass index, *LTPA* leisure-time physical activity, *CHD* coronary heart disease, *h/d* hours per day, min/wk min per week, *NHANES* National Health and Nutrition Examination Survey

^a^Weighted to be nationally representative. Weighted percentage may not sum to 100% because of missing data

^b^Including American Indian/Alaska Native/Pacific Islander, Asian, and multiracial

^C^Refers to the year in which the participants were enrolled in NHANES

### Relationship between sitting time, vitamin D, and mortality

During up to 13.2 years (median, 5.6 years), 676 deaths occurred, including 226 deaths from cancer, 142 from cardiovascular disease, and 308 from other causes. The multivariable-adjusted model (Table 2) revealed that cancer survivors who had a daily sitting time of more than 8 h/day had a higher risk of all-cause mortality (HR, 1.71; 95% CI, 1.33–2.20), noncancer mortality (HR, 1.94; 95% CI, 1.42–2.66), and a marginally higher risk of cancer mortality (HR, 1.38; 95% CI, 0.90–2.11) than those with sitting time less than 4 h/day. Additionally, each 1 h/day increase in daily sitting time was associated with a 6%, 3%, and 8% increase in the risk of all-cause, cancer, and noncancer mortality, respectively. Moreover, vitamin D deficiency was associated with a significantly higher risk of all-cause mortality (HR, 1.46; 95% CI, 1.18–1.81) and cancer mortality (HR, 1.89; 95% CI, 1.34–2.67) than those without vitamin D deficiency. A 25 nmol/L increase in vitamin D level was associated with a 14%, 18%, and 12% decrease in the risk of all-cause, cancer, and noncancer mortality, respectively (Additional file 2: Fig. S1).

**Table 2:** Association of daily sitting time and vitamin D status with all-cause, cancer, and noncancer mortality among US cancer survivors, NHANES, 2007 to 2018

			**Hazard ratio (95% CI), *P* value**					
**Study outcome**	**Death/No**	**Weighted death (%)**	**Age adjusted**^**a**^		**MV model 1**^**b**^		**MV model 2**^**c**^	
All-cause mortality	676/2914	21,542,524 (16.5)						
Daily sitting time, h/day								
< 4	178/980	6,702,663 (12.9)	1 [Reference]		1 [Reference]		1 [Reference]	
4 to < 6	168/733	5,328,337 (16.6)	1.10 (0.89, 1.36)	0.381	1.01 (0.80, 1.28)	0.928	1.01 (0.80, 1.28)	0.930
6 to 8	157/570	4,161,260 (19.1)	1.48 (1.19, 1.83)	< 0.001	1.35 (1.06, 1.72)	0.014	1.33 (1.04, 1.69)	0.022
> 8	173/631	5,350,265 (18.8)	1.77 (1.43, 2.18)	< 0.001	1.73 (1.35, 2.23)	< 0.001	1.71 (1.33, 2.20)	< 0.001
Per 1 h/day increase			1.07 (1.04, 1.09)	< 0.001	1.06 (1.04, 1.09)	< 0.001	1.06 (1.03, 1.09)	< 0.001
Vitamin D status								
≥ 50 nmol/L (non-deficiency)	506/2348	18,440,705 (15.1)	1 [Reference]		1 [Reference]		1 [Reference]	
< 50 nmol/L (deficiency)	170/566	3,101,819 (24.4)	1.67 (1.40, 1.99)	< 0.001	1.49 (1.20, 1.85)	< 0.001	1.46 (1.18, 1.81)	0.001
Per 25 nmol/L increase			0.83 (0.77, 0.89)	< 0.001	0.86 (0.79, 0.93)	< 0.001	0.86 (0.79, 0.93)	< 0.001
Cancer mortality	226/2915	21,546,130 (5.7)						
Daily sitting time, h/day								
< 4	67/980	6,706,269 (5.0)	1 [Reference]		1 [Reference]		1 [Reference]	
4 to < 6	59/733	5,328,337 (6.0)	1.09 (0.77, 1.55)	0.620	0.94 (0.63, 1.39)	0.752	0.93 (0.63, 1.38)	0.731
6 to 8	50/570	4,161,260 (6.2)	1.33 (0.92, 1.92)	0.131	1.20 (0.80, 1.80)	0.377	1.18 (0.79, 1.77)	0.425
> 8	50/631	5,350,265 (5.8)	1.39 (0.96, 2.01)	0.081	1.39 (0.91, 2.13)	0.133	1.38 (0.90, 2.11)	0.144
Per 1 h/day increase			1.04 (0.99, 1.08)	0.086	1.03 (0.98, 1.08)	0.212	1.03 (0.98, 1.08)	0.237
Vitamin D status								
≥ 50 nmol/L (non-deficiency)	160/2348	18,440,705 (5.0)	1 [Reference]		1 [Reference]		1 [Reference]	
< 50 nmol/L (deficiency)	66/566	3,105,425 (9.8)	1.93 (1.44, 2.57)	< 0.001	1.89 (1.34, 2.67)	< 0.001	1.89 (1.34, 2.67)	< 0.001
Per 25 nmol/L increase			0.81 (0.71, 0.91)	0.001	0.82 (0.71, 0.95)	0.009	0.82 (0.71, 0.95)	0.008
Noncancer mortality	450/2915	21,546,130 (10.8)						
Daily sitting time, h/day								
< 4	111/980	6,702,663 (7.9)	1 [Reference]		1 [Reference]		1 [Reference]	
4 to < 6	109/733	5,328,337 (10.6)	1.11 (0.85, 1.45)	0.444	1.05 (0.78, 1.41)	0.736	1.06 (0.79, 1.42)	0.707
6 to 8	107/570	4,161,260 (12.9)	1.57 (1.20, 2.05)	0.001	1.45 (1.07, 1.95)	0.016	1.42 (1.05, 1.92)	0.022
> 8	123/631	5,350,265 (13.0)	2.00 (1.54, 2.59)	< 0.001	1.97 (1.44, 2.70)	< 0.001	1.94 (1.42, 2.66)	< 0.001
Per 1 h/day increase			1.08 (1.05, 1.11)	< 0.001	1.08 (1.05, 1.12)	< 0.001	1.08 (1.05, 1.12)	< 0.001
Vitamin D status								
≥ 50 nmol/L (non-deficiency)	346/2348	18,440,705 (10.2)	1 [Reference]		1 [Reference]		1 [Reference]	
< 50 nmol/L (deficiency)	104/566	3,105,425 (14.7)	1.54 (1.23, 1.91)	< 0.001	1.28 (0.97, 1.69)	0.081	1.24 (0.94, 1.64)	0.126
Per 25 nmol/L increase			0.84 (0.77, 0.91)	< 0.001	0.88 (0.79, 0.97)	0.012	0.88 (0.79, 0.97)	0.012

^a^Adjusted for age

^b^Multivariable adjusted model additionally adjusted for sex, race and ethnicity, educational attainment, baseline year, family poverty income ratio, body mass index, physical activity, smoking status, and alcohol use

^c^Additionally adjusted for hypertension, diabetes, and coronary heart disease

### Joint association of sitting time and vitamin D with mortality

**Fig. 3:**
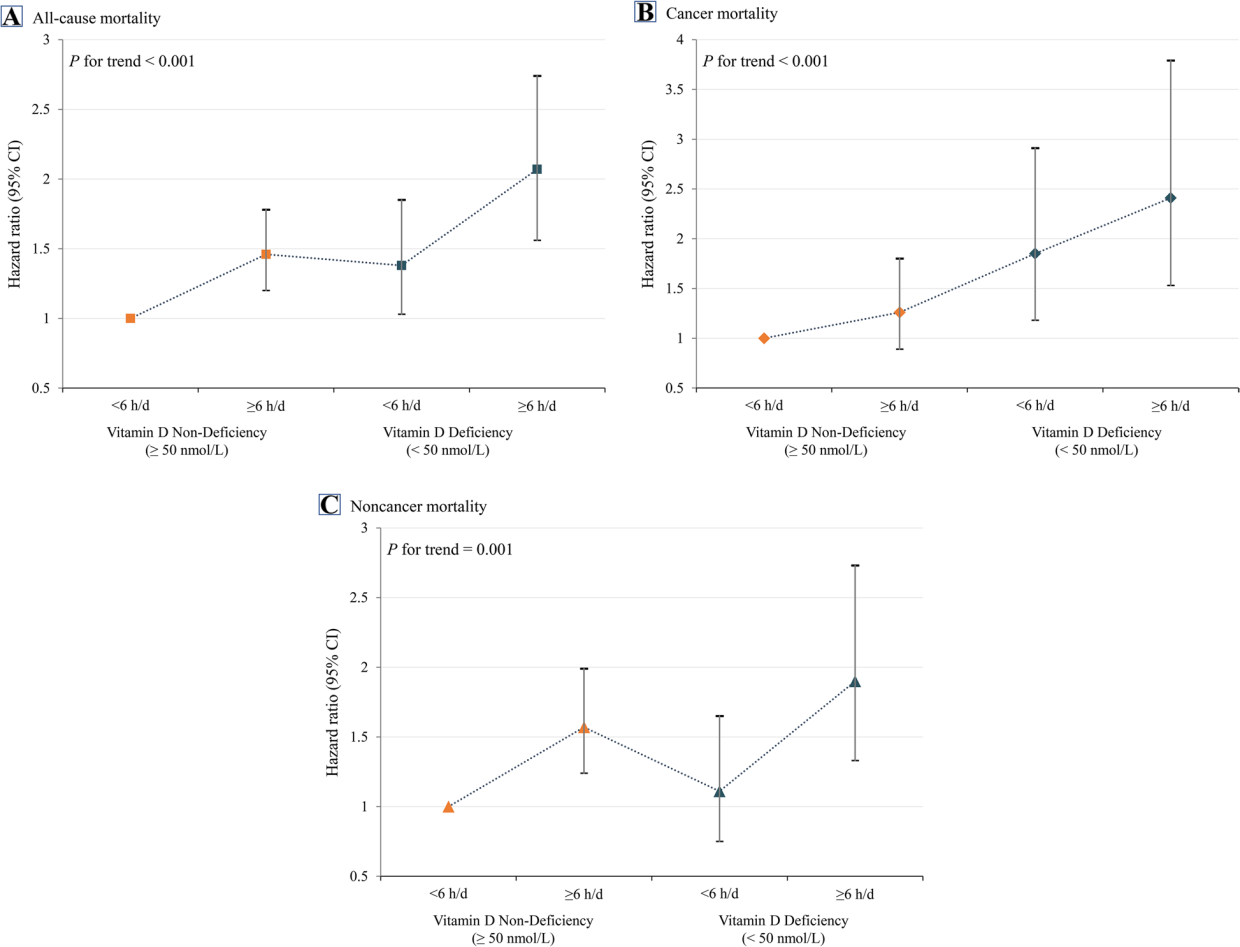
Joint association of daily sitting time and vitamin D status with all-cause (**A**), cancer (**B**), and noncancer (**C**) mortality in a nationally representative sample of US cancer survivors, NHANES 2007 to 2018. The solid symbols and error bars represent the hazard ratios and their corresponding 95% confidence interval. Adjusted for age, sex, race and ethnicity, educational attainment, baseline year, family poverty income ratio, body mass index, physical activity, smoking status, alcohol use, hypertension, diabetes, and coronary heart disease

In joint analyses, cancer survivors with vitamin D deficiency and sedentary behavior (≥ 6 h/day) had the highest risk of all-cause, cancer, and noncancer mortality (Fig. 3). Compared to the combination of vitamin D non-deficiency and sitting time less than 6 h/day, the HRs for all-cause, cancer, and noncancer in the groups of vitamin D deficiency and sitting time ≥ 6 h/day were 2.05 (1.54, 2.72), 2.33 (1.47, 3.70), and 1.91 (1.33, 2.74), respectively (Table 3). A sensitivity analysis was conducted to evaluate the robustness of our primary results. Upon excluding patients with less than 3 years of follow-up time, the findings remained consistent with our main results (Additional file 2: Table S1).

**Table 3:** Joint association of daily sitting time and vitamin D status with all-cause, cancer, and noncancer mortality among US cancer survivors, NHANES, 2007 to 2018

				**Hazard ratio (95% CI), *P *value**					
**Study outcome**	**Sitting time, h/d**	**Death/No**	**Weighted death (%)**	**Age adjusted**^**a**^		**MV model 1**^**b**^		**MV model 2**^**c**^	
**All-cause mortality**									
Vitamin D non-deficiency	< 6	268/1401	10485267 (13.8)	1 [Reference]		1 [Reference]		1 [Reference]	
(≥ 50 nmol/L)	≥ 6	238/947	7955439 (16.9)	1.48 (1.24, 1.76)	< 0.001	1.45 (1.19, 1.77)	0.001	1.43 (1.17, 1.74)	0.001
Vitamin D deficiency	< 6	78/312	1545733 (19.7)	1.54 (1.20, 1.99)	0.001	1.40 (1.04, 1.88)	0.027	1.38 (1.02, 1.85)	0.036
(< 50 nmol/L)	≥ 6	92/254	1556086 (29.1)	2.54 (2.00, 3.22)	< 0.001	2.13 (1.60, 2.82)	< 0.001	2.05 (1.54, 2.72)	< 0.001
*P* for trend test				< 0.001		< 0.001		< 0.001	
**Cancer mortality**									
Vitamin D non-deficiency	< 6	93/1401	10485267 (5.1)	1 [Reference]		1 [Reference]		1 [Reference]	
(≥ 50 nmol/L)	≥ 6	67/947	7955439 (4.8)	1.23 (0.89, 1.68)	0.207	1.25 (0.88, 1.79)	0.218	1.23 (0.86, 1.76)	0.260
Vitamin D Deficiency	< 6	33/312	1549339 (7.9)	1.80 (1.20, 2.68)	0.004	1.83 (1.16, 2.88)	0.010	1.80 (1.14, 2.84)	0.012
(< 50 nmol/L)	≥ 6	33/254	1556086 (11.6)	2.49 (1.67, 3.71)	< 0.001	2.34 (1.48, 3.70)	0.0003	2.33 (1.47, 3.70)	< 0.001
*P* for trend test				< 0.001		< 0.001		< 0.001	
**Noncancer mortality**									
Vitamin D non-deficiency	< 6	175/1401	10485267 (8.7)	1 [Reference]		1 [Reference]		1 [Reference]	
(≥ 50 nmol/L)	≥ 6	171/947	7955439 (12.1)	1.61 (1.30, 1.99)	< 0.001	1.55 (1.22, 1.97)	< 0.001	1.53 (1.20, 1.95)	0.001
Vitamin D deficiency	< 6	45/312	1549339 (11.8)	1.39 (1.00, 1.94)	0.049	1.14 (0.76, 1.69)	0.530	1.12 (0.75, 1.66)	0.581
(< 50 nmol/L)	≥ 6	59/254	1556086 (17.5)	2.58 (1.91, 3.47)	< 0.001	2.02 (1.41, 2.90)	< 0.001	1.91 (1.33, 2.74)	0.001
*P* for trend test				< 0.001		< 0.001		0.001	

^a^Adjusted for age

^b^Multivariable adjusted model additionally adjusted for sex, race and ethnicity, educational attainment, baseline year, family poverty income ratio, body mass index, physical activity, smoking status, and alcohol use

^c^Additionally adjusted for hypertension, diabetes, and coronary heart disease

### Interaction test

Our results indicated significant interaction on both the additive and multiplicative scales. The RERI was 0.38 for all-cause mortality, 0.46 for cancer mortality, and 0.33 for noncancer mortality, suggesting the joint effect of sedentary behavior and vitamin D status exceeds the sum of their independent effects on all-cause cancer, and noncancer mortality. The ROR was 1.17 for all-cause mortality, 1.21 for cancer mortality, and 1.18 for noncancer mortality (Additional file 1: Table S2). This indicates that the joint effect of sedentary behavior and vitamin D deficiency is greater than expected under a multiplicative model.

### Subgroup analysis

In age-stratified analyses, the association between sedentary behavior, vitamin D deficiency, and mortality was more pronounced in cancer survivors under 65 years than in those 65 and older (Additional file 2: Table S2). The joint effect of vitamin D and sedentary time on mortality was consistent across age groups (Additional file 2: Table S3). In sex-stratified analyses, prolonged sitting time had a greater adverse effect in women, while the negative association between vitamin D and mortality was more evident in men (Additional file 2: Table S4). These combined associations remained stable across sex subgroups (Additional file 2: Table S5). In the stratified analysis by cancer type, distinct variations were observed in the prevalence of both sedentary behavior and vitamin D deficiency. Specifically, sedentary behavior (> 6 h/day) was more prevalent among survivors of respiratory system tumors (45.9%), vitamin D deficiency was notably higher among survivors of gastrointestinal tumors (33.9%) (Additional file 2: Table S6). The combined adverse effects of vitamin D deficiency and sedentary behavior on mortality are significantly evident in cancer survivors with gynecological malignancies, male urological malignancies, urological malignancies, and skin cancer. However, no such joint deleterious effects were observed in survivors of other cancer subtypes (Additional file 2: Table S7). In subgroup analyses stratified by PA levels, these associations persisted in the subgroup with insufficient physical activity (< 150 min/week). However, in the subgroup with sufficient physical activity (≥ 150 min/week), the relationships became non-statistically significant (Additional file 2: Table S8). In subgroup analyses stratified by baseline year, these relationships were generally consistent in both subgroups (2007–2012 and 2013–2018). Interestingly, the adverse combined effect on all-cause mortality and cancer mortality was more pronounced in the subgroup with baseline years 2013–2018 (Additional file 2: Table S9). In stratified analyses of smoking categories, we identified a dose–response association with smoking status. Importantly, when examining the interaction between vitamin D levels and sedentary behavior, a graded increase in mortality risk was observed with escalating smoking intensity. Specifically, current smokers with a history exceeding 10 pack-years exhibited the highest mortality risk (Additional file 2: Table S10 & Fig. S2).

## Discussion

This study is the first to investigate the combined effect of daily sitting time and vitamin D status on survival in a US nationally representative cancer population. Among these cancer survivors, 62.7% had a daily sitting time of more than 6 h, and 14.4% had vitamin D deficiency. Over the 13.2-year follow-up period, prolonged sitting time was associated with an increased risk of all-cause and non-cancer mortality, while vitamin D deficiency was associated with a higher risk of all-cause and cancer mortality. In the joint analysis, vitamin D-deficient cancer survivors with prolonged sitting time (> 6 h/day) had the higher risk of all-cause, cancer, and noncancer mortality than those with a single risk factor.

Prolonged sitting can be detrimental for cancer survivors as it may promote tumor growth, reduce the effectiveness of anticancer treatments, and contribute to poor prognosis [34]. A study by Cao et al. [4] found that prolonged sitting time was independently associated with a higher risk of all-cause and cancer mortality but not non-cancer mortality in cancer populations. However, several questions remain to be explored. Firstly, Cao et al. limited the age of the study population to over 40 years, which may have introduced a selection bias as aging is an independent risk factor for poor survival [35]. In contrast, our study included cancer survivors of all ages and found that prolonged sedentary time was associated with an increased risk of all-cause and non-cancer mortality, but not cancer mortality. This discrepancy might be because the positive relationship between sedentary behavior and cancer mortality was significant only when sitting time exceeded a certain threshold (> 8 h/day). Additionally, younger cancer survivors with prolonged sedentary time were more likely to die from noncancer-related causes, such as neurocognitive dysfunction, cardiovascular disease, and psychosocial problems [36, 37]. Secondly, the adverse effects on survival of decreased physical activity and prolonged sedentary time are similar, which may limit their contribution to developing treatment strategies [38]. From this perspective, a joint investigation of differential risk factors could provide a more integrative understanding of the survival status of cancer survivors, which motivated us to conduct this new study.

Vitamin D deficiency is a potential risk factor for poor survival due to its anti-inflammatory and immune-modulating effects [39]. Studies investigating the association between vitamin D deficiency and all-cause and cause-specific mortality have yielded inconsistent results, particularly concerning cancer-related deaths. Chowdhury et al. [40] conducted a meta-analysis that reported a link between low vitamin D concentrations and increased risk of all-cause, cardiovascular, cancer, and non-cancer mortality, leading to the possibility of vitamin D supplementation as a preventive strategy for cancer and improved survival. However, Zhang Y et al. [41] conducted a subsequent meta-analysis that demonstrated a 15% reduction in cancer mortality risk with vitamin D supplementation but found no association with all-cause mortality. In contrast, Manson et al. [12] reported no reduction in all-cause, cancer and cardiovascular mortality risk with vitamin D supplementation in a randomized controlled trial (RCT). Recently, a UK biobank study indicated an L-shaped relationship between vitamin D concentrations and all-cause, cardiovascular, and cancer mortality, with an inflection point at 50 nmol/L [42]. We observed a negative association between vitamin D and all-cause and specific mortality only in the low range of vitamin D concentrations, and increasing vitamin D concentration failed to provide additional survival benefits. Our study may explain the inconsistency between observational studies and RCTs, as large observational cohorts are more likely to include those with low vitamin D concentrations, whereas maintaining participants in long-term vitamin D deficiency status in an RCT may not be ethical or feasible [12].

In this study, we chose to conduct a joint analysis of sedentary behavior and vitamin D status, as opposed to examining the interaction term in the full model. This decision was based on the complex interactions between these variables, which may not be adequately captured by a simple interaction term. Both sedentary behavior and vitamin D deficiency have been independently associated with adverse health outcomes, including increased mortality in cancer survivors [9, 43]. Furthermore, our study provides compelling evidence of a significant joint association between sedentary behavior and vitamin D status with mortality among cancer survivors. The significance of the interaction term on both the additive and multiplicative scales support the hypothesis that cancer survivors who both engage in prolonged sitting and are vitamin D deficient are at particularly high risk for mortality. The joint analysis allows us to explore the unique and combined contributions of each variable to mortality outcomes, providing a more comprehensive understanding of the survival status of cancer survivors [44, 45]. Specifically, our findings indicated that cancer survivors with vitamin D deficiency and sedentary behavior (> 6 h/day) had significantly higher risks of all-cause, cancer, and non-cancer mortality than those with only one risk factor. Importantly, the consistent findings after excluding individuals with less than 3 years of follow-up time add robustness to our main conclusions and alleviate concerns about reverse causation. The lack of significant changes in our results after this exclusion suggests that our original findings were not significantly influenced by individuals who were severely ill or close to death at baseline.

Our findings demonstrate the importance of considering cancer type in evaluating the joint impact of sedentary behavior and vitamin D status on survival outcomes. For instance, the significant association observed among gynecologic tumors survivors may be partly explained by the detrimental effects of sedentary behavior on estrogen metabolism [46]. The absence of a significant association among gastrointestinal tumors survivors could be related to other lifestyle factors not accounted for in the study, like diet or medication use [47]. These variations underscore the need for individualized lifestyle recommendations based on cancer type. Additionally, the adverse effects of sedentary behavior and vitamin D deficiency on survival in cancer survivors persisted even after adjustment for PA, highlighting the unique nature of sedentary behavior and its potential health implications. Notably, the associations were more pronounced among those with insufficient physical activity, suggesting that meeting recommended physical activity levels might mitigate the potential risks associated with prolonged sedentary behavior and vitamin D deficiency [30]. This attenuation of risk in the physically active subgroup resonates with existing literature emphasizing the protective effects of regular exercise against various adverse health outcomes [48].

The stratification of the study population into two subgroups based on baseline years did not significantly influence the primary outcomes. This implies that the observed associations remain consistent despite potential temporal variations in cancer treatment and healthcare accessibility during the study duration. Nevertheless, the adverse effects were more pronounced in the later enrollment period (2013–2018). Such a trend might be due to advances in cancer treatment, underscoring a heightened interaction between vitamin D status and sedentary behavior in the backdrop of enhanced overall survival rates. Additionally, this may suggest that lifestyle factors have gained increased significance in shaping outcomes in recent years. Our enhanced categorization of smoking status has provided deeper insights into its dose–response relationship with mortality. This granularity has underscored the significance of smoking as a critical confounder in our analysis. Past research has shown the detrimental effects of smoking on cancer survivorship [49]. By detailing smoking habits, our study furthers the understanding of its impact on mortality in this specific cohort, underscoring the importance of comprehensive smoking cessation programs for cancer survivors.

Our findings have several possible biological explanations. Sedentary behavior is associated with increased risks of obesity, metabolic syndrome, diabetes, and CVD, which can negatively impact long-term health and survival [50, 51]. Additionally, prolonged sitting time can weaken immune function and promote tumor growth by increasing systemic inflammation [52]. Similarly, vitamin D has several antitumor effects, including inhibiting tumor cell proliferation, regulating cell death, inhibiting angiogenesis, and modulating the immune system and inflammation [53, 54]. Therefore, vitamin D deficiency and sedentary behavior may have similar adverse biological effects on cancer survivor survival, which could explain the combined effect of these two risk factors.

## Strengths and limitations

The study’s strengths include its use of a representative sample of US cancer survivors, as well as a multiethnic population, increasing the generalizability of the findings to other cancer populations. Additionally, the study conducted several sensitivity analyses to assess the robustness of the results. Despite this, there are a few limitations to be considered. First, one of the major limitations of our study is the reliance on self-reported cancer diagnoses. Although some studies have indicated generally good agreement between self-reported cancer history and medical records, self-reported data may be subject to recall bias and misclassification [23, 24]. Future studies with direct access to medical records or cancer registry data could provide more definitive conclusions. Second, the study relied on self-reported data for sitting time and physical activity. While these methods have shown moderate to good reliability in past studies, they are still susceptible to biases such as recall bias. Objective measures, like accelerometry, can provide more accurate data, though they come with their own set of challenges. Future studies should consider integrating both self-reported and objective data for a more comprehensive understanding. Third, the study measured daily sitting time and vitamin D concentrations at baseline, and dynamic changes in these factors were not collected during the follow-up. Finally, the study’s observational nature means that the effects of residual confounders and unknowns cannot be completely ruled out in the study analysis.

## Conclusions

Our study, conducted on a representative cohort of U.S. cancer survivors, revealed a significant association between extended daily sedentary time and vitamin D deficiency. Furthermore, we observed that cancer survivors who exhibited both vitamin D deficiency and sedentary behavior faced an elevated risk of all-cause mortality, as well as cancer-specific and non-cancer mortality. These results underscore the importance of considering both sedentary behavior and vitamin D status in developing targeted intervention strategies to improve survival outcomes among cancer survivors.

### Supplementary Information

**Additional file 1: Table S1. **Smoking status was classified into five categories. **Table S2. **Interaction test for the relationship of vitamin D status and sedentary behavior with mortality. **Fig. S1. **Directed acyclic plot (DAG) between exposure factors (sedentary behavior, vitamin D deficiency), mortality and covariates.

**Additional file 2: Fig. S1. **Dose-response association of daily sitting time and vitamin D status with all-cause, cancer, and noncancer mortality among US cancer survivors. **Fig. S2. **Association of combined vitamin D status and sedentary time with all-cause mortality in smoking categories. **Table S1. **Association between daily sitting time and vitamin D levels with mortality (excluding follow-ups shorter than 3 years). **Table S2. **Association of daily sitting time and vitamin D status with all-cause mortality among US cancer survivors stratified by age (<65, ≥65 years). **Table S3. **Joint association of daily sitting time and vitamin D status with all-cause mortality among US cancer survivors stratified by age (<65, ≥65 years). **Table S4. **Association of daily sitting time and vitamin D status with all-cause mortality among US cancer survivors stratified by sex (male, female). **Table S5. **Joint association of daily sitting time and vitamin D status with all-cause mortality among US cancer survivors stratified by sex (male, female). **Table S6. **Baseline characteristics of US cancer survivors and stratified by nine categories of cancer. **Table S7. **Association of daily sitting time and vitamin D status with all-cause mortality in survivors with various cancer types. **Table S8. **Independent and joint association of daily sitting time and vitamin D status with mortality stratified by LTPA. **Table S9. **Independent and joint association of daily sitting time and vitamin D status with mortality stratified by baseline year.** Table S10. **Association of combined daily sitting time and vitamin D status with all-cause mortality in smoking categories.

## Data Availability

The datasets generated and analyzed in the current study are available at NHANES website: https://www.cdc.gov/nchs/nhanes/index.htm.
