# Supplementary material for: Joint association of sedentary behavior and vitamin D status with mortality among cancer survivors
Source: BMC Med. 2023 Oct 31;21:411. doi: 10.1186/s12916-023-03118-9 (PMC10617233; doi:10.1186/s12916-023-03118-9)
Supplement: Supplementary file 1 — Additional file 1: Table S1. Smoking status was classified into five categories. Table S2. Interaction test for the relationship of vitamin D status and sedentary behavior with mortality. Fig. S1. Directed acyclic plot (DAG) between exposure factors (sedentary behavior, vitamin D deficiency), mortality and covariates. [file 12916_2023_3118_MOESM1_ESM.docx]

**Supplementary methods**

**Smoking status**

| **Table S1.** Smoking status was classified into five categories | |
| --- | --- |
| Never smoked | Individuals who have smoked fewer than 100 cigarettes in their lifetime |
| Former smoker, light | <10 pack-years |
| Former smoker, heavy | ≥10 pack-years |
| Current smoker, light | <10 pack-years |
| Current smoker, heavy | ≥10 pack-years |

Pack-years will be calculated by multiplying the number of packs of cigarettes smoked per day by the number of years the person has smoked.

**Calculation of RERI and ROR**

To calculate additive and multiplicative effects, we performed an interaction test on the relationship of vitamin D status and sedentary behavior with mortality, as shown below:

| **Table S2.** Interaction test for the relationship of vitamin D status and sedentary behavior with mortality | | | |
| --- | --- | --- | --- |
| **Exposure** |  | **HR (95% CI)** | **P value** |
| **All-cause mortality** |  |  |  |
| *Vitamin D Status* | *Sitting time* |  |  |
| ≥50 nmol/L | <6 h/d | Ref. |  |
| <50 nmol/L (Deficiency) | <6 h/d | 1.18 (0.92, 1.52) | 0.19 |
| ≥50 nmol/L | ≥6 h/d | 1.49 (1.25, 1.78) | <0.001 |
| <50 nmol/L (Deficiency) | ≥6 h/d | 2.05 (1.62, 2.60) | <0.001 |
| P interaction |  | 0.401 |  |
| **Cancer mortality** |  |  |  |
| *Vitamin D Status* | *Sitting time* |  |  |
| ≥50 nmol/L | <6 h/d | Ref. |  |
| <50 nmol/L (Deficiency) | <6 h/d | 1.45 (0.98, 2.16) | 0.066 |
| ≥50 nmol/L | ≥6 h/d | 1.20 (0.87, 1.64) | 0.26 |
| <50 nmol/L (Deficiency) | ≥6 h/d | 2.11 (1.42, 3.14) | <0.001 |
| P interaction |  | 0.51 |  |
| **Noncancer mortality** |  |  |  |
| *Vitamin D Status* | *Sitting time* |  |  |
| ≥50 nmol/L | <6 h/d | Ref. |  |
| <50 nmol/L (Deficiency) | <6 h/d | 1.04 (0.75, 1.45) | 0.801 |
| ≥50 nmol/L | ≥6 h/d | 1.65 (1.34, 2.04) | <0.001 |
| <50 nmol/L (Deficiency) | ≥6 h/d | 2.02 (1.50, 2.71) | <0.001 |
| P interaction |  | 0.479 |  |

**1. Additive Scale Interaction:** We have computed the relative excess risk due to interaction (RERI) to capture interaction on the additive scale. RERI elucidates whether the joint effect of sedentary behavior and vitamin D status exceeds the sum of their independent effects.

The RERI is generally calculated using the following formula: RERI=OR_11_-OR_10_-OR_01_+1

Where: OR_11_ is the Odds Ratio for subjects with both risk factors (sedentary behavior and low Vitamin D status), OR_10_ is the Odds Ratio for subjects with the first risk factor (sedentary behavior) but not the second (low Vitamin D status), OR_01_ is the Odds Ratio for subjects with the second risk factor (low Vitamin D status) but not the first (sedentary behavior).

RERI > 0 indicates a positive additive interaction, RERI < 0 indicates a negative additive interaction, and RERI = 0 indicates no additive interaction.

The RERI was 0.38 for all-cause mortality, 0.46 for cancer mortality, and 0.33 for noncancer mortality.

**2. Multiplicative Scale Interaction:** For the multiplicative scale, we will incorporate the ratio of odds ratios (ROR) to evaluate the interaction on the multiplicative scale. This will indicate whether the joint effect of sedentary behavior and Vitamin D deficiency is different from what would be expected based on their independent effects multiplied together.

Calculating Ratio of Odds Ratios (ROR): ROR= OR_11_/ (OR_10_×OR_01_)

If ROR>1, the combined effect of both factors is greater than expected under a multiplicative model; If ROR<1, the combined effect of both factors is less than expected.

The ROR was 1.17 for all-cause mortality, 1.21 for cancer mortality, and 1.18 for noncancer mortality.

**Confounder Selection and Model Building:**

Confounding variables were selected based on three criteria: clinical relevance, a P-value less than 0.05 in univariate analysis, and the availability of sufficient event data to construct a robust regression model. Clinical relevance was established by employing a multifaceted approach that included Directed Acyclic Graphs (DAGs), comprehensive literature reviews, and consultations with subject-matter experts. DAGs, a graphical representation of the causal relationships among variables, aided in highlighting potential confounders in the association between sedentary behavior, vitamin D status, and mortality in cancer survivors, as shown in the following figure. Further insights from existing literature and expert opinion ensured a comprehensive list of confounders. *Exposures and Outcome Relationship*: Both Vitamin D and Sedentary Behavior are the exposures of interest. We hypothesize that these exposures have a direct effect on the mortality. Adjustment Variables: *Age, Sex, Race, Education, Family income*: These are demographic variables. Age is a well-known confounder as both the exposure (Vitamin D levels, Sedentary Behavior) and outcome (Mortality) could be influenced by age. The same reasoning can be applied for sex, race, education, and family income. *Baseline Years*: Reflects the period of enrollment which can influence both exposure and outcome due to the evolving nature of cancer care over time. *BMI*: Can be affected by sedentary behavior and can also affect Vitamin D metabolism. Furthermore, BMI can influence mortality risk. *Physical Activity*: Sedentary behavior and physical activity are related, and both can affect mortality. *Smoking and Drinking*: Both can influence mortality. Additionally, lifestyle factors like smoking and drinking can influence one's likelihood of engaging in sedentary behavior. *Hypertension, Diabetes, CHD*: These comorbid conditions can both be influenced by our exposures and directly affect mortality.


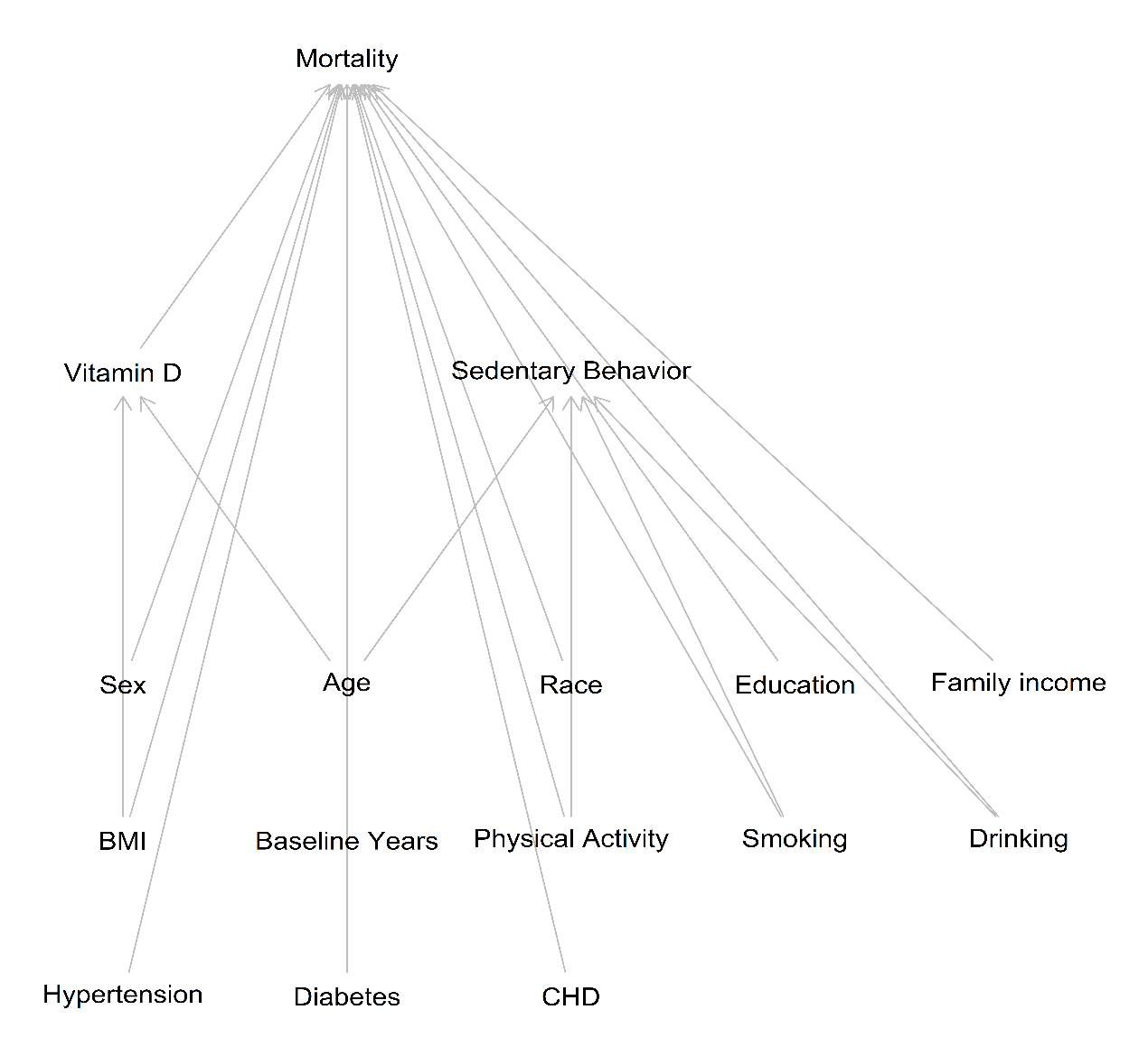


**Fig. S1.** Directed acyclic plot (DAG) between exposure factors (sedentary behavior, vitamin D deficiency), mortality and covariates
