# Supplementary material for: Joint association of sedentary behavior and vitamin D status with mortality among cancer survivors
Source: BMC Med. 2023 Oct 31;21:411. doi: 10.1186/s12916-023-03118-9 (PMC10617233; doi:10.1186/s12916-023-03118-9)
Supplement: Supplementary file 2 — Additional file 2: Fig. S1. Dose-response association of daily sitting time and vitamin D status with all-cause, cancer, and noncancer mortality among US cancer survivors. Fig. S2. Association of combined vitamin D status and sedentary time with all-cause mortality in smoking categories. Table S1. Association between daily sitting time and vitamin D levels with mortality (excluding follow-ups shorter than 3 years). Table S2. Association of daily sitting time and vitamin D status with all-cause mortality among US cancer survivors stratified by age (<65, ≥65 years). Table S3. Joint association of daily sitting time and vitamin D status with all-cause mortality among US cancer survivors stratified by age (<65, ≥65 years). Table S4. Association of daily sitting time and vitamin D status with all-cause mortality among US cancer survivors stratified by sex (male, female). Table S5. Joint association of daily sitting time and vitamin D status with all-cause mortality among US cancer survivors stratified by sex (male, female). Table S6. Baseline characteristics of US cancer survivors and stratified by nine categories of cancer. Table S7. Association of daily sitting time and vitamin D status with all-cause mortality in survivors with various cancer types. Table S8. Independent and joint association of daily sitting time and vitamin D status with mortality stratified by LTPA. Table S9. Independent and joint association of daily sitting time and vitamin D status with mortality stratified by baseline year. Table S10. Association of combined daily sitting time and vitamin D status with all-cause mortality in smoking categories. [file 12916_2023_3118_MOESM2_ESM.docx]

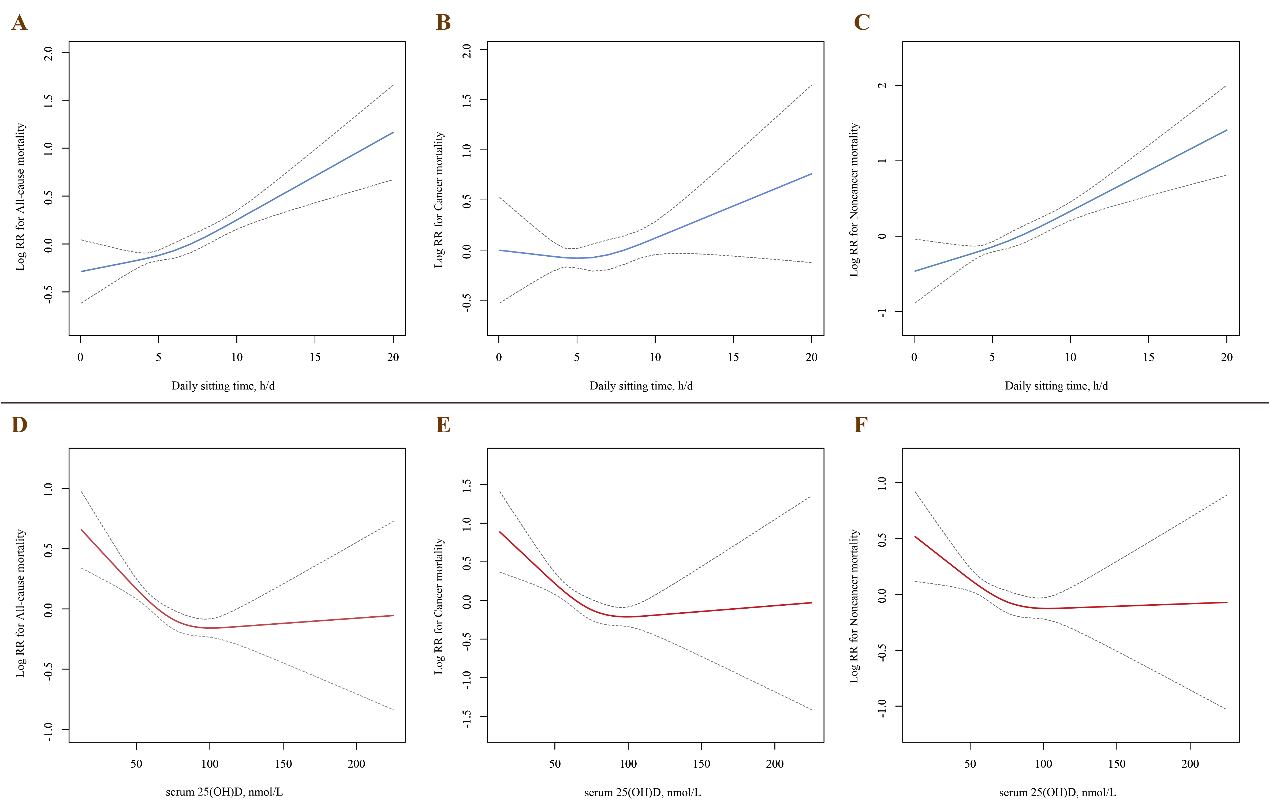


**Fig. S1.** Dose-response association of daily sitting time and vitamin D status with all-cause, cancer, and noncancer mortality among US cancer survivors

The solid line and dashed line represent the estimated hazard ratios and their corresponding 95% confidence interval.

A, B, and C indicate the relationship between sedentary time and all-cause, cancer, and non-cancer mortality; D, E, and F represent the relationship between serum 25(OH)D and all-cause, cancer, and non-cancer mortality, respectively.

Adjusted for age, sex, race and ethnicity, educational attainment, baseline year, family poverty income ratio, body mass index, physical activity, smoking status, alcohol use, hypertension, diabetes, and coronary heart disease.

| **Table S1.** Association between daily sitting time and vitamin D levels with mortality (excluding follow-ups shorter than 3 years) | | | | | | |
| --- | --- | --- | --- | --- | --- | --- |
|  | **Hazard ratio (95% CI), P value** | | |  |  |  |
| Exposure | **Age adjusted^a^** |  | **MV model 1^b^** |  | **MV model 2^c^** |  |
| **All-cause mortality** |  |  |  |  |  |  |
| Vitamin D Non-Deficiency | 1 [Reference] |  | 1 [Reference] |  | 1 [Reference] |  |
| (≥50 nmol/L) | 1.43 (1.15, 1.79) | 0.001 | 1.54 (1.21, 1.96) | <0.001 | 1.52 (1.19, 1.93) | 0.001 |
| Vitamin D Deficiency | 1.35 (0.98, 1.84) | 0.064 | 1.20 (0.83, 1.75) | 0.334 | 1.19 (0.82, 1.73) | 0.366 |
| (<50 nmol/L) | 2.44 (1.80, 3.30) | <0.001 | 2.30 (1.63, 3.25) | <0.001 | 2.18 (1.54, 3.09) | <0.001 |
| P for trend test | <0.001 |  | <0.001 |  | <0.001 |  |
| **Cancer mortality** |  |  |  |  |  |  |
| Vitamin D Non-Deficiency | 1 [Reference] |  | 1 [Reference] |  | 1 [Reference] |  |
| (≥50 nmol/L) | 1.21 (0.81, 1.82) | 0.346 | 1.45 (0.92, 2.28) | 0.108 | 1.44 (0.91, 2.26) | 0.117 |
| Vitamin D Deficiency | 1.17 (0.66, 2.06) | 0.595 | 1.24 (0.65, 2.39) | 0.512 | 1.23 (0.64, 2.36) | 0.537 |
| (<50 nmol/L) | 1.74 (0.97, 3.11) | 0.064 | 2.04 (1.08, 3.87) | 0.029 | 1.99 (1.05, 3.79) | 0.036 |
| P for trend test | 0.085 |  | 0.032 |  | 0.04 |  |
| **Noncancer mortality** |  |  |  |  |  |  |
| Vitamin D Non-Deficiency | 1 [Reference] |  | 1 [Reference] |  | 1 [Reference] |  |
| (≥50 nmol/L) | 1.54 (1.18, 2.00) | 0.001 | 1.58 (1.19, 2.09) | 0.002 | 1.55 (1.16, 2.06) | 0.003 |
| Vitamin D Deficiency | 1.43 (0.98, 2.09) | 0.062 | 1.16 (0.73, 1.84) | 0.517 | 1.15 (0.73, 1.82) | 0.555 |
| (<50 nmol/L) | 2.81 (1.97, 4.01) | <0.001 | 2.42 (1.61, 3.65) | <0.001 | 2.25 (1.49, 3.40) | <0.001 |
| P for trend test | <0.001 |  | <0.001 |  | <0.001 |  |

*^a^ Adjusted for age.*

*^b^ Multivariable adjusted model additionally adjusted for sex, race and ethnicity, educational attainment, baseline year, family poverty income ratio, body mass index, physical activity, smoking status, and alcohol use.*

*^c^ Additionally adjusted for hypertension, diabetes, and coronary heart disease.*

| **Table S2.** Association of daily sitting time and vitamin D status with all-cause mortality among US cancer survivors stratified by age (<65, ≥65 years) | | | | | | | | |
| --- | --- | --- | --- | --- | --- | --- | --- | --- |
|  |  |  | **Hazard ratio (95% CI), P value** | |  |  |  |  |
| **Subgroup** | **Death/No.** | **Weighted death (%)** | **Age adjusted^a^** |  | **MV model 1^b^** |  | **MV model 2^c^** |  |
| **Age <65 years** | 115/1234 | 6.1 |  |  |  |  |  |  |
| Daily sitting time, h/d |  |  |  |  |  |  |  |  |
| <4 | 35/478 | 4.9 | 1 [Reference] |  | 1 [Reference] |  | 1 [Reference] |  |
| 4 to <6 | 27/257 | 6.5 | 1.38 (0.84, 2.28) | 0.208 | 1.66 (0.97, 2.86) | 0.067 | 1.71 (0.99, 2.95) | 0.053 |
| 6 to 8 | 27/222 | 8.2 | 1.73 (1.05, 2.86) | 0.033 | 1.60 (0.92, 2.77) | 0.094 | 1.50 (0.86, 2.61) | 0.153 |
| >8 | 26/277 | 5.9 | 1.46 (0.88, 2.43) | 0.147 | 2.13 (1.21, 3.75) | 0.009 | 2.24 (1.27, 3.96) | 0.006 |
| Per 1 h/d increase |  |  | 1.05 (0.99, 1.10) | 0.087 | 1.08 (1.02, 1.14) | 0.012 | 1.08 (1.02, 1.15) | 0.008 |
| Vitamin D Status |  |  |  |  |  |  |  |  |
| ≥50 nmol/L (Non-Deficiency) | 62/920 | 4.7 | 1 [Reference] |  | 1 [Reference] |  | 1 [Reference] |  |
| <50 nmol/L (Deficiency) | 53/314 | 12.9 | 2.74 (1.90, 3.96) | <0.001 | 2.19 (1.42, 3.38) | <0.001 | 2.03 (1.31, 3.14) | 0.001 |
| Per 25 nmol/L increase |  |  | 0.62 (0.52, 0.75) | <0.001 | 0.71 (0.57, 0.87) | 0.001 | 0.72 (0.58, 0.88) | 0.002 |
| **Age ≥65 years** | 561/1680 | 27.5 |  |  |  |  |  |  |
| Daily sitting time, h/d |  |  |  |  |  |  |  |  |
| <4 | 143/502 | 23.6 | 1 [Reference] |  | 1 [Reference] |  | 1 [Reference] |  |
| 4 to <6 | 141/476 | 23.9 | 1.06 (0.84, 1.34) | 0.626 | 0.92 (0.71, 1.20) | 0.549 | 0.92 (0.71, 1.19) | 0.514 |
| 6 to 8 | 130/348 | 28.5 | 1.44 (1.13, 1.83) | 0.003 | 1.28 (0.98, 1.67) | 0.065 | 1.26 (0.96, 1.64) | 0.090 |
| >8 | 147/354 | 36.1 | 1.86 (1.47, 2.35) | <0.001 | 1.79 (1.36, 2.35) | <0.001 | 1.74 (1.32, 2.28) | <0.001 |
| Per 1 h/d increase |  |  | 1.07 (1.05, 1.10) | <0.001 | 1.07 (1.04, 1.11) | <0.001 | 1.07 (1.04, 1.10) | <0.001 |
| Vitamin D Status |  |  |  |  |  |  |  |  |
| ≥50 nmol/L (Non-Deficiency) | 444/1428 | 25.5 | 1 [Reference] |  | 1 [Reference] |  | 1 [Reference] |  |
| <50 nmol/L (Deficiency) | 117/252 | 42.2 | 1.44 (1.17, 1.77) | 0.001 | 1.30 (1.01, 1.68) | 0.041 | 1.27 (0.99, 1.64) | 0.062 |
| Per 25 nmol/L increase |  |  | 0.87 (0.81, 0.94) | <0.001 | 0.91 (0.83, 1.00) | 0.041 | 0.91 (0.83, 1.00) | 0.047 |

^a^ Adjusted for age.

^b^ Multivariable adjusted model additionally adjusted for sex, race and ethnicity, educational attainment, baseline year, family poverty income ratio, body mass index, physical activity, smoking status, and alcohol use.

^c^ Additionally adjusted for hypertension, diabetes, and coronary heart disease.

| **Table S3.** Joint association of daily sitting time and vitamin D status with all-cause mortality among US cancer survivors stratified by age (<65, ≥65 years) | | | | | | | | | |
| --- | --- | --- | --- | --- | --- | --- | --- | --- | --- |
|  |  |  |  | **Hazard ratio (95% CI), P value** | | |  |  |  |
| **Subgroup** | **Sitting time, h/d** | **Death/No.** | **Weighted death (%)** | **Age adjusted^a^** |  | **MV model 1^b^** |  | **MV model 2^c^** |  |
| **Age <65 years** |  |  |  |  |  |  |  |  |  |
| Vitamin D Non-Deficiency | <6 | 34/552 | 4.8 | 1 [Reference] |  | 1 [Reference] |  | 1 [Reference] |  |
| (≥50 nmol/L) | ≥6 | 28/368 | 4.5 | 1.42 (0.86, 2.35) | 0.170 | 1.69 (0.97, 2.92) | 0.062 | 1.66 (0.95, 2.88) | 0.074 |
| Vitamin D Deficiency | <6 | 28/183 | 9.2 | 2.90 (1.75, 4.80) | <0.001 | 2.50 (1.42, 4.41) | 0.002 | 2.33 (1.32, 4.12) | 0.004 |
| (<50 nmol/L) | ≥6 | 25/131 | 16.6 | 3.54 (2.11, 5.94) | <0.001 | 2.97 (1.63, 5.40) | <0.001 | 2.72 (1.48, 4.99) | 0.001 |
| P for trend test |  |  |  | <0.001 |  | <0.001 |  | <0.001 |  |
| **Age ≥65 years** |  |  |  |  |  |  |  |  |  |
| Vitamin D Non-Deficiency | <6 | 234/849 | 22.4 | 1 [Reference] |  | 1 [Reference] |  | 1 [Reference] |  |
| (≥50 nmol/L) | ≥6 | 210/579 | 29.8 | 1.49 (1.24, 1.80) | <0.001 | 1.47 (1.19, 1.81) | <0.001 | 1.43 (1.16, 1.77) | 0.001 |
| Vitamin D Deficiency | <6 | 50/129 | 35.9 | 1.24 (0.91, 1.68) | 0.175 | 1.11 (0.77, 1.61) | 0.570 | 1.08 (0.75, 1.57) | 0.664 |
| (<50 nmol/L) | ≥6 | 67/123 | 48.3 | 2.36 (1.79, 3.09) | <0.001 | 2.05 (1.48, 2.85) | <0.001 | 1.98 (1.42, 2.75) | <0.001 |
| P for trend test |  |  |  | <0.001 |  | <0.001 |  | <0.001 |  |

^a^ Adjusted for age.

^b^ Multivariable adjusted model additionally adjusted for sex, race and ethnicity, educational attainment, baseline year, family poverty income ratio, body mass index, physical activity, smoking status, and alcohol use.

^c^ Additionally adjusted for hypertension, diabetes, and coronary heart disease.

| **Table S4.** Association of daily sitting time and vitamin D status with all-cause mortality among US cancer survivors stratified by sex (male, female) | | | | | | | | |
| --- | --- | --- | --- | --- | --- | --- | --- | --- |
|  |  |  | **Hazard ratio (95% CI), P value** | | |  |  |  |
| **Subgroup** | **Death/No.** | **Weighted death (%)** | **Age adjusted^a^** |  | **MV model 1^b^** |  | **MV model 2^c^** |  |
| **Men** | 405/1381 | 20.5 |  |  |  |  |  |  |
| Daily sitting time, h/d |  |  |  |  |  |  |  |  |
| <4 | 116/446 | 18.3 | 1 [Reference] |  | 1 [Reference] |  | 1 [Reference] |  |
| 4 to <6 | 99/343 | 21.8 | 1.05 (0.80, 1.38) | 0.708 | 1.03 (0.77, 1.38) | 0.824 | 1.01 (0.76, 1.35) | 0.938 |
| 6 to 8 | 94/289 | 20.5 | 1.35 (1.02, 1.78) | 0.033 | 1.31 (0.97, 1.77) | 0.077 | 1.27 (0.94, 1.72) | 0.121 |
| >8 | 98/303 | 22 | 1.58 (1.20, 2.07) | 0.001 | 1.68 (1.22, 2.30) | 0.002 | 1.62 (1.17, 2.22) | 0.003 |
| Per 1 h/d increase |  |  | 1.05 (1.02, 1.08) | 0.003 | 1.06 (1.02, 1.10) | 0.002 | 1.06 (1.02, 1.09) | 0.003 |
| Vitamin D Status |  |  |  |  |  |  |  |  |
| ≥50 nmol/L (Non-Deficiency) | 313/1140 | 19.4 | 1 [Reference] |  | 1 [Reference] |  | 1 [Reference] |  |
| <50 nmol/L (Deficiency) | 94/241 | 27.7 | 1.67 (1.32, 2.12) | <0.001 | 1.68 (1.26, 2.23) | <0.001 | 1.66 (1.24, 2.21) | 0.001 |
| Per 25 nmol/L increase | |  | 0.83 (0.75, 0.92) | <0.001 | 0.82 (0.73, 0.93) | 0.002 | 0.82 (0.72, 0.92) | 0.001 |
| **Women** | 269/1533 | 13.3 |  |  |  |  |  |  |
| Daily sitting time, h/d |  |  |  |  |  |  |  |  |
| <4 | 62/534 | 9.2 | 1 [Reference] |  | 1 [Reference] |  | 1 [Reference] |  |
| 4 to <6 | 69/390 | 12.9 | 1.21 (0.86, 1.71) | 0.271 | 1.00 (0.67, 1.47) | 0.981 | 1.01 (0.68, 1.50) | 0.948 |
| 6 to 8 | 63/281 | 17.7 | 1.67 (1.17, 2.38) | 0.0045 | 1.44 (0.96, 2.16) | 0.075 | 1.43 (0.95, 2.14) | 0.084 |
| >8 | 75/328 | 16.1 | 2.07 (1.47, 2.91) | <0.001 | 2.00 (1.35, 2.98) | 0.0006 | 2.01 (1.35, 2.99) | 0.0006 |
| Per 1 h/d increase |  |  | 1.09 (1.05, 1.13) | <0.001 | 1.09 (1.04, 1.13) | <0.001 | 1.08 (1.04, 1.13) | <0.001 |
| Vitamin D Status |  |  |  |  |  |  |  |  |
| ≥50 nmol/L (Non-Deficiency) | 193/1208 | 11.7 | 1 [Reference] |  | 1 [Reference] |  | 1 [Reference] |  |
| <50 nmol/L (Deficiency) | 76/325 | 22.3 | 1.69 (1.30, 2.21) | <0.001 | 1.30 (0.94, 1.80) | 0.112 | 1.22 (0.88, 1.69) | 0.234 |
| Per 25 nmol/L increase | |  | 0.83 (0.75, 0.92) | <0.001 | 0.91 (0.81, 1.02) | 0.102 | 0.92 (0.82, 1.03) | 0.161 |

^a^ Adjusted for age.

^b^ Multivariable adjusted model additionally adjusted for sex, race and ethnicity, educational attainment, baseline year, family poverty income ratio, body mass index, physical activity, smoking status, and alcohol use.

^c^ Additionally adjusted for hypertension, diabetes, and coronary heart disease.

| **Table S5.** Joint association of daily sitting time and vitamin D status with all-cause mortality among US cancer survivors stratified by sex (male, female) | | | | | | | | | |
| --- | --- | --- | --- | --- | --- | --- | --- | --- | --- |
|  |  |  |  | **Hazard ratio (95% CI), P value** | | |  |  |  |
| **Subgroup** | **Sitting time, h/d** | **Death/No.** | **Weighted death (%)** | **Age adjusted^a^** |  | **MV model 1^b^** |  | **MV model 2^c^** |  |
| **Men** |  |  |  |  |  |  |  |  |  |
| Vitamin D Non-Deficiency | <6 | 174/673 | 19.5 | 1 [Reference] |  | 1 [Reference] |  | 1 [Reference] |  |
| (> 50 nmol/L) | >6 | 139/467 | 19.4 | 1.35 (1.08, 1.69) | 0.009 | 1.37 (1.06, 1.76) | 0.015 | 1.32 (1.03, 1.71) | 0.030 |
| Vitamin D Deficiency | <6 | 41/116 | 23 | 1.51 (1.07, 2.14) | 0.018 | 1.57 (1.05, 2.34) | 0.027 | 1.53 (1.03, 2.29) | 0.036 |
| (< 50 nmol/L) | >6 | 53/125 | 31.1 | 2.35 (1.72, 3.22) | <0.001 | 2.19 (1.52, 3.14) | <0.001 | 2.14 (1.49, 3.08) | <0.001 |
| P for trend test |  |  |  | <0.001 |  | <0.001 |  | <0.001 |  |
| **Women** |  |  |  |  |  |  |  |  |  |
| Vitamin D Non-Deficiency | <6 | 94/728 | 9.6 | 1 [Reference] |  | 1 [Reference] |  | 1 [Reference] |  |
| (> 50 nmol/L) | >6 | 99/480 | 14.7 | 1.69 (1.27, 2.24) | <0.001 | 1.74 (1.26, 2.40) | 0.001 | 1.76 (1.27, 2.43) | 0.001 |
| Vitamin D Deficiency | <6 | 37/196 | 18.1 | 1.70 (1.16, 2.49) | 0.007 | 1.36 (0.86, 2.14) | 0.190 | 1.34 (0.85, 2.11) | 0.213 |
| (< 50 nmol/L) | >6 | 39/129 | 27.5 | 2.81 (1.93, 4.09) | <0.001 | 2.14 (1.35, 3.38) | 0.001 | 1.91 (1.21, 3.03) | 0.006 |
| P for trend test |  |  |  | <0.001 |  | 0.001 |  | 0.005 |  |

^a^ Adjusted for age.

^b^ Multivariable adjusted model additionally adjusted for sex, race and ethnicity, educational attainment, baseline year, family poverty income ratio, body mass index, physical activity, smoking status, and alcohol use.

^c^ Additionally adjusted for hypertension, diabetes, and coronary heart disease.

| **Table S6.** Baseline characteristics of US cancer survivors and stratified by nine categories of cancer | | | | | | | | | |
| --- | --- | --- | --- | --- | --- | --- | --- | --- | --- |
| *Characteristic* | **Gynecologic Tumors** | **Urologic Tumors (Male)** | **Head and Neck Tumors** | **Respiratory System Tumors** | **Gastrointestinal Tumors** | **Urologic Tumors** | **Skin Cancer** | **Hematologic Malignancies** | **Other Cancers** |
| Participant | 792 | 463 | 88 | 61 | 248 | 129 | 843 | 99 | 167 |
| Age, y | 60.6 ± 15.1 | 71.0 ± 9.1 | 59.6 ± 14.5 | 69.0 ± 11.2 | 68.9 ± 11.8 | 70.9 ± 10.6 | 67.4 ± 13.0 | 56.2 ± 18.8 | 60.7 ± 16.1 |
| Sex |  |  |  |  |  |  |  |  |  |
| Men | 0 (0.0%) | 463 (100.0%) | 32 (36.4%) | 35 (57.4%) | 128 (51.6%) | 96 (74.4%) | 476 (56.5%) | 57 (57.6%) | 87 (52.1%) |
| Women | 792 (100.0%) | 0 (0.0%) | 56 (63.6%) | 26 (42.6%) | 120 (48.4%) | 33 (25.6%) | 367 (43.5%) | 42 (42.4%) | 80 (47.9%) |
| Race and ethnicity | |  |  |  |  |  |  |  |  |
| Non-Hispanic White | 455 (57.4%) | 235 (50.8%) | 49 (55.7%) | 35 (57.4%) | 146 (58.9%) | 87 (67.4%) | 780 (92.5%) | 56 (56.6%) | 101 (60.5%) |
| Hispanic | 172 (21.7%) | 52 (11.2%) | 19 (21.6%) | 4 (6.6%) | 36 (14.5%) | 16 (12.4%) | 41 (4.9%) | 19 (19.2%) | 28 (16.8%) |
| Non-Hispanic Black | 109 (13.8%) | 148 (32.0%) | 13 (14.8%) | 14 (23.0%) | 53 (21.4%) | 19 (14.7%) | 7 (0.8%) | 15 (15.2%) | 21 (12.6%) |
| Other^b^ | 56 (7.1%) | 28 (6.0%) | 7 (8.0%) | 8 (13.1%) | 13 (5.2%) | 7 (5.4%) | 15 (1.8%) | 9 (9.1%) | 17 (10.2%) |
| Sitting time, h/d | 6.2 ± 3.4 | 6.3 ± 3.4 | 5.9 ± 3.4 | 6.7 ± 3.0 | 6.3 ± 3.4 | 6.5 ± 3.3 | 6.6 ± 3.1 | 6.4 ± 3.2 | 6.4 ± 3.4 |
| <6 | 472 (59.6%) | 282 (60.9%) | 58 (65.9%) | 33 (54.1%) | 148 (59.7%) | 71 (55.0%) | 477 (56.6%) | 59 (59.6%) | 97 (58.1%) |
| >6 | 320 (40.4%) | 181 (39.1%) | 30 (34.1%) | 28 (45.9%) | 100 (40.3%) | 58 (45.0%) | 366 (43.4%) | 40 (40.4%) | 70 (41.9%) |
| Vitamin D, nmol/L | |  |  |  |  |  |  |  |  |
| ≥50 (non-Deficiency) | 609 (76.9%) | 364 (78.6%) | 75 (85.2%) | 46 (75.4%) | 164 (66.1%) | 109 (84.5%) | 755 (89.6%) | 72 (72.7%) | 137 (82.0%) |
| <50 (Deficiency) | 183 (23.1%) | 99 (21.4%) | 13 (14.8%) | 15 (24.6%) | 84 (33.9%) | 20 (15.5%) | 88 (10.4%) | 27 (27.3%) | 30 (18.0%) |
| LTPA, min/wk | |  |  |  |  |  |  |  |  |
| ≥150 (Active) | 180 (61.4%) | 150 (69.8%) | 23 (62.2%) | 10 (45.5%) | 46 (56.8%) | 32 (71.1%) | 293 (71.1%) | 36 (75.0%) | 60 (80.0%) |
| <150 (Inactive) | 113 (38.6%) | 65 (30.2%) | 14 (37.8%) | 12 (54.5%) | 35 (43.2%) | 13 (28.9%) | 119 (28.9%) | 12 (25.0%) | 15 (20.0%) |
| Follow-up, years | 6.6 ± 3.5 | 5.8 ± 3.3 | 6.2 ± 3.4 | 4.8 ± 3.4 | 5.5 ± 3.5 | 5.2 ± 3.3 | 6.3 ± 3.4 | 6.1 ± 3.8 | 5.7 ± 3.5 |
| Mortality | 124 (15.7%) | 128 (27.6%) | 15 (17.0%) | 25 (41.0%) | 92 (37.1%) | 41 (31.8%) | 180 (21.4%) | 24 (24.2%) | 46 (27.5%) |

| **Table S7.** Association of daily sitting time and vitamin D status with all-cause mortality in survivors with various cancer types | | | |
| --- | --- | --- | --- |
|  | **Hazard ratio (95% CI)** | |  |
|  | **Gynecologic Tumors** | **Urologic Tumors (Male)** | **Head and Neck Tumors** |
| **Daily sitting time, h/d** |  |  |  |
| <4 | 1 | 1 | 1 |
| 4 to <6 | 1.27 (0.75, 2.14) | 1.32 (0.81, 2.17) | 0.64 (0.13, 3.17) |
| 6 to 8 | 1.50 (0.89, 2.55) | 2.10 (1.29, 3.42) ** | 0.49 (0.06, 4.16) |
| >8 | 2.34 (1.43, 3.82) *** | 2.21 (1.36, 3.60) ** | 3.07 (0.81, 11.57) |
| **Vitamin D Status** |  |  |  |
| ≥50 nmol/L (Non-Deficiency) | 1 | 1 | 1 |
| <50 nmol/L (Deficiency) | 1.71 (1.16, 2.51) ** | 1.36 (0.91, 2.04) | 8.18 (1.99, 33.60) ** |
| **Vitamin D status & Sitting time** |  |  |  |
| NonDeficiency, <6 hr/d | 1 | 1 | 1 |
| NonDeficiency, ≥6 hr/d | 1.65 (1.07, 2.54) * | 1.72 (1.14, 2.59) ** | 2.64 (0.73, 9.49) |
| Deficiency, <6 hr/d | 1.62 (0.92, 2.84) | 1.10 (0.61, 1.98) | 14.73 (2.59, 83.79) ** |
| Deficiency, ≥6 hr/d | 3.15 (1.83, 5.43) *** | 3.01 (1.73, 5.23) *** | 9.99 (0.98, 102.24) |
|  | **Respiratory System Tumors** | **Gastrointestinal Tumors** | **Urologic Tumors** |
| **Daily sitting time, h/d** |  |  |  |
| <4 | 1 | 1 | 1 |
| 4 to <6 | 1.37 (0.35, 5.41) | 0.99 (0.56, 1.74) | 1.60 (0.58, 4.40) |
| 6 to 8 | 2.07 (0.49, 8.70) | 1.31 (0.74, 2.33) | 2.22 (0.93, 5.33) |
| >8 | 2.17 (0.48, 9.87) | 1.66 (0.92, 2.99) | 2.43 (1.02, 5.82) * |
| **Vitamin D Status** |  |  |  |
| ≥50 nmol/L (Non-Deficiency) | 1 | 1 | 1 |
| <50 nmol/L (Deficiency) | 2.17 (0.81, 5.80) | 1.35 (0.88, 2.07) | 2.93 (1.44, 5.95) ** |
| **Vitamin D status & Sitting time** |  |  |  |
| NonDeficiency, <6 hr/d | 1 | 1 | 1 |
| NonDeficiency, ≥6 hr/d | 1.69 (0.61, 4.73) | 1.78 (1.02, 3.10) * | 1.90 (0.91, 3.97) |
| Deficiency, <6 hr/d | 2.69 (0.51, 14.26) | 1.67 (0.92, 3.03) | 3.02 (0.94, 9.64) |
| Deficiency, ≥6 hr/d | 2.95 (0.83, 10.50) | 1.62 (0.91, 2.87) | 4.99 (1.93, 12.88) *** |
|  | **Skin Cancer** | **Hematologic Tumors** | **Other Cancers** |
| **Daily sitting time, h/d** |  |  |  |
| <4 | 1 | 1 | 1 |
| 4 to <6 | 0.77 (0.51, 1.16) | 1.67 (0.60, 4.65) | 1.09 (0.47, 2.52) |
| 6 to 8 | 1.08 (0.70, 1.66) | 1.08 (0.29, 4.02) | 1.49 (0.66, 3.38) |
| >8 | 1.25 (0.82, 1.90) | 0.67 (0.17, 2.72) | 1.90 (0.85, 4.28) |
| **Vitamin D Status** |  |  |  |
| ≥50 nmol/L (Non-Deficiency) | 1 | 1 | 1 |
| <50 nmol/L (Deficiency) | 1.29 (0.84, 1.98) | 2.66 (0.91, 7.75) | 3.17 (1.48, 6.79) ** |
| **Vitamin D status & Sitting time** |  |  |  |
| NonDeficiency, <6 hr/d | 1 | 1 | 1 |
| NonDeficiency, ≥6 hr/d | 1.30 (0.94, 1.79) | 0.46 (0.15, 1.45) | 1.88 (0.97, 3.66) |
| Deficiency, <6 hr/d | 1.13 (0.59, 2.18) | 2.41 (0.67, 8.59) | 8.25 (2.64, 25.81) *** |
| Deficiency, ≥6 hr/d | 1.79 (1.01, 3.16) * | 2.04 (0.50, 8.25) | 3.02 (1.09, 8.42) * |

*Adjusted for age, sex, race and ethnicity, educational attainment, baseline year, family poverty income ratio, body mass index, physical activity, smoking status, alcohol use*

*hypertension, diabetes, and coronary heart disease.*

| **Table S8.** Independent and joint association of daily sitting time and vitamin D status with mortality stratified by LTPA | | | | |
| --- | --- | --- | --- | --- |
|  | **LTPA>150 min/wk (Active)** | | **LTPA<150 min/wk (Inactive)** | |
|  | **HR (95% CI)** | **P value** | **HR (95% CI)** | **P value** |
| **All-cause mortality** |  |  |  |  |
| Daily sitting time, h/d |  |  |  |  |
| <4 | 1 [Reference] |  | 1 [Reference] |  |
| 4 to <6 | 1.06 (0.65, 1.72) | 0.816 | 0.99 (0.76, 1.30) | 0.947 |
| 6 to 8 | 1.06 (0.62, 1.82) | 0.833 | 1.40 (1.07, 1.84) | 0.014 |
| >8 | 1.23 (0.64, 2.35) | 0.541 | 1.86 (1.42, 2.44) | <0.001 |
| Vitamin D Status |  |  |  |  |
| ≥50 nmol/L (Non-Deficiency) | 1 [Reference] |  | 1 [Reference] |  |
| <50 nmol/L (Deficiency) | 1.09 (0.57, 2.11) | 0.790 | 1.46 (1.16, 1.83) | 0.001 |
| **Vitamin D Status & Sitting Time** |  |  |  |  |
| NonDeficiency, <6 hr/d | 1 [Reference] |  | 1 [Reference] |  |
| NonDeficiency, >6 hr/d | 1.01 (0.65, 1.58) | 0.961 | 1.61 (1.28, 2.01) | <0.001 |
| Deficiency, <6 hr/d | 0.84 (0.34, 2.09) | 0.704 | 1.45 (1.06, 2.00) | 0.021 |
| Deficiency, >6 hr/d | 1.49 (0.62, 3.56) | 0.368 | 2.15 (1.59, 2.91) | <0.001 |
| **Cancer mortality** |  |  |  |  |
| Daily sitting time, h/d |  |  |  |  |
| <4 | 1 [Reference] |  | 1 [Reference] |  |
| 4 to <6 | 0.71 (0.27, 1.84) | 0.475 | 0.98 (0.64, 1.52) | 0.943 |
| 6 to 8 | 1.15 (0.44, 2.97) | 0.777 | 1.19 (0.76, 1.86) | 0.440 |
| >8 | 1.86 (0.69, 5.01) | 0.221 | 1.30 (0.81, 2.08) | 0.271 |
| Vitamin D Status |  |  |  |  |
| ≥50 nmol/L (Non-Deficiency) | 1 [Reference] |  | 1 [Reference] |  |
| <50 nmol/L (Deficiency) | 1.54 (0.57, 4.17) | 0.397 | 1.90 (1.31, 2.74) | 0.001 |
| **Vitamin D Status & Sitting Time** |  |  |  |  |
| NonDeficiency, <6 hr/d | 1 [Reference] |  | 1 [Reference] |  |
| NonDeficiency, >6 hr/d | 1.48 (0.67, 3.26) | 0.326 | 1.20 (0.80, 1.79) | 0.379 |
| Deficiency, <6 hr/d | 1.21 (0.31, 4.74) | 0.782 | 1.87 (1.15, 3.04) | 0.012 |
| Deficiency, >6 hr/d | 2.72 (0.73, 10.06) | 0.135 | 2.22 (1.36, 3.62) | 0.002 |
| **Noncancer mortality** |  |  |  |  |
| Daily sitting time, h/d |  |  |  |  |
| <4 | 1 [Reference] |  | 1 [Reference] |  |
| 4 to <6 | 1.26 (0.71, 2.23) | 0.431 | 0.99 (0.70, 1.39) | 0.9553 |
| 6 to 8 | 1.02 (0.53, 1.99) | 0.948 | 1.56 (1.11, 2.19) | 0.0104 |
| >8 | 0.93 (0.38, 2.26) | 0.875 | 2.29 (1.63, 3.21) | <0.001 |
| Vitamin D Status |  |  |  |  |
| ≥50 nmol/L (Non-Deficiency) | 1 [Reference] |  | 1 [Reference] |  |
| <50 nmol/L (Deficiency) | 0.89 (0.36, 2.17) | 0.791 | 1.25 (0.93, 1.67) | 0.1394 |
| **Vitamin D Status & Sitting Time** |  |  |  |  |
| NonDeficiency, <6 hr/d | 1 [Reference] |  | 1 [Reference] |  |
| NonDeficiency, >6 hr/d | 0.84 (0.48, 1.46) | 0.538 | 1.88 (1.43, 2.47) | <0.001 |
| Deficiency, <6 hr/d | 0.69 (0.20, 2.45) | 0.568 | 1.21 (0.79, 1.84) | 0.3819 |
| Deficiency, >6 hr/d | 1.03 (0.31, 3.50) | 0.957 | 2.15 (1.46, 3.17) | <0.001 |

*Adjusted for age, sex, race and ethnicity, educational attainment, baseline year, family poverty income ratio, body mass index, smoking status, alcohol use*

*hypertension, diabetes, and coronary heart disease.*

| **Table S9.** Independent and joint association of daily sitting time and vitamin D status with mortality stratified by baseline year | | | | |
| --- | --- | --- | --- | --- |
|  | **Baseline year (2007-2012)** | | **Baseline year (2013-2018)** | |
|  | **HR (95% CI)** | **P value** | **HR (95% CI)** | **P value** |
| **All-cause mortality** |  |  |  |  |
| Daily sitting time, h/d |  |  |  |  |
| <4 | 1 [Reference] |  | 1 [Reference] |  |
| 4 to <6 | 1.07 (0.83, 1.37) | 0.588 | 1.06 (0.66, 1.69) | 0.813 |
| 6 to 8 | 1.49 (1.15, 1.93) | 0.002 | 1.38 (0.87, 2.18) | 0.169 |
| >8 | 1.63 (1.24, 2.15) | 0.001 | 1.95 (1.27, 3.02) | 0.003 |
| Vitamin D Status |  |  |  |  |
| ≥50 nmol/L (Non-Deficiency) | 1 [Reference] |  | 1 [Reference] |  |
| <50 nmol/L (Deficiency) | 1.47 (1.16, 1.85) | 0.001 | 1.87 (1.31, 2.66) | 0.001 |
| **Vitamin D Status & Sitting Time** |  |  |  |  |
| NonDeficiency, <6 hr/d | 1 [Reference] |  | 1 [Reference] |  |
| NonDeficiency, >6 hr/d | 1.44 (1.16, 1.80) | 0.001 | 1.67 (1.19, 2.33) | 0.003 |
| Deficiency, <6 hr/d | 1.37 (1.00, 1.86) | 0.048 | 2.40 (1.33, 4.34) | 0.004 |
| Deficiency, >6 hr/d | 2.14 (1.56, 2.92) | <0.001 | 2.44 (1.54, 3.86) | <0.001 |
| **Cancer mortality** |  |  |  |  |
| Daily sitting time, h/d |  |  |  |  |
| <4 | 1 [Reference] |  | 1 [Reference] |  |
| 4 to <6 | 1.08 (0.71, 1.63) | 0.734 | 0.93 (0.44, 1.96) | 0.858 |
| 6 to 8 | 1.34 (0.85, 2.09) | 0.205 | 1.34 (0.66, 2.70) | 0.420 |
| >8 | 1.40 (0.87, 2.27) | 0.168 | 1.51 (0.75, 3.02) | 0.250 |
| Vitamin D Status |  |  |  |  |
| ≥50 nmol/L (Non-Deficiency) | 1 [Reference] |  | 1 [Reference] |  |
| <50 nmol/L (Deficiency) | 1.59 (1.07, 2.36) | 0.023 | 2.82 (1.63, 4.87) | <0.001 |
| **Vitamin D Status & Sitting Time** |  |  |  |  |
| NonDeficiency, <6 hr/d | 1 [Reference] |  | 1 [Reference] |  |
| NonDeficiency, >6 hr/d | 1.26 (0.84, 1.86) | 0.261 | 1.50 (0.83, 2.74) | 0.181 |
| Deficiency, <6 hr/d | 1.49 (0.90, 2.49) | 0.124 | 3.38 (1.48, 7.74) | 0.004 |
| Deficiency, >6 hr/d | 2.02 (1.19, 3.45) | 0.010 | 3.50 (1.69, 7.29) | 0.001 |
| **Noncancer mortality** |  |  |  |  |
| Daily sitting time, h/d |  |  |  |  |
| <4 | 1 [Reference] |  | 1 [Reference] |  |
| 4 to <6 | 1.07 (0.79, 1.46) | 0.664 | 1.16 (0.63, 2.13) | 0.634 |
| 6 to 8 | 1.58 (1.15, 2.16) | 0.005 | 1.44 (0.78, 2.65) | 0.242 |
| >8 | 1.76 (1.25, 2.47) | 0.001 | 2.29 (1.30, 4.03) | 0.004 |
| Vitamin D Status |  |  |  |  |
| ≥50 nmol/L (Non-Deficiency) | 1 [Reference] |  | 1 [Reference] |  |
| <50 nmol/L (Deficiency) | 1.41 (1.05, 1.88) | 0.021 | 1.38 (0.85, 2.24) | 0.188 |
| **Vitamin D Status & Sitting Time** |  |  |  |  |
| NonDeficiency, <6 hr/d | 1 [Reference] |  | 1 [Reference] |  |
| NonDeficiency, >6 hr/d | 1.54 (1.17, 2.02) | 0.002 | 1.76 (1.17, 2.65) | 0.007 |
| Deficiency, <6 hr/d | 1.30 (0.88, 1.92) | 0.188 | 1.67 (0.69, 4.07) | 0.259 |
| Deficiency, >6 hr/d | 2.19 (1.49, 3.22) | <0.001 | 1.93 (1.06, 3.50) | 0.031 |

*Adjusted for age, sex, race and ethnicity, educational attainment, family poverty income ratio, body mass index, physical activit, smoking status, alcohol use*

*hypertension, diabetes, and coronary heart disease.*

| **Table S10.** Association of combined daily sitting time and vitamin D status with all-cause mortality in smoking categories | | |
| --- | --- | --- |
|  | **Hazard ratio (95% CI)** | **P value** |
| **Never smoked** |  |  |
| NonDeficiency, <6 hr/d | 1 [Reference] |  |
| NonDeficiency, >6 hr/d | 1.42 (1.06, 1.90) | 0.019 |
| Deficiency, <6 hr/d | 1.27 (0.82, 1.98) | 0.290 |
| Deficiency, >6 hr/d | 2.57 (1.71, 3.85) | <0.001 |
| **Former smoker, light** |  |  |
| NonDeficiency, <6 hr/d | 0.78 (0.51, 1.19) | 0.248 |
| NonDeficiency, >6 hr/d | 1.51 (1.01, 2.27) | 0.044 |
| Deficiency, <6 hr/d | 2.22 (1.07, 4.60) | 0.031 |
| Deficiency, >6 hr/d | 2.13 (0.93, 4.87) | 0.073 |
| **Former smoker, heavy** |  |  |
| NonDeficiency, <6 hr/d | 1.50 (1.13, 1.99) | 0.005 |
| NonDeficiency, >6 hr/d | 2.23 (1.68, 2.95) | <0.001 |
| Deficiency, <6 hr/d | 2.10 (1.30, 3.40) | 0.002 |
| Deficiency, >6 hr/d | 3.59 (2.43, 5.30) | <0.001 |
| **Current smoker, light** |  |  |
| NonDeficiency, <6 hr/d | 2.56 (1.59, 4.12) | <0.001 |
| NonDeficiency, >6 hr/d | 2.98 (1.49, 5.94) | 0.002 |
| Deficiency, <6 hr/d | 2.96 (1.60, 5.49) | 0.001 |
| Deficiency, >6 hr/d | 2.89 (1.25, 6.68) | 0.013 |
| **Current smoker, heavy** |  |  |
| NonDeficiency, <6 hr/d | 2.61 (1.60, 4.28) | <0.001 |
| NonDeficiency, >6 hr/d | 5.07 (2.68, 9.60) | <0.001 |
| Deficiency, <6 hr/d | 5.50 (2.99, 10.09) | <0.001 |
| Deficiency, >6 hr/d | 5.72 (3.23, 10.14) | <0.001 |

*Adjusted for age, sex, race and ethnicity, baseline year, educational attainment, family poverty income ratio, body mass index, physical activit, alcohol use*

*hypertension, diabetes, and coronary heart disease.*


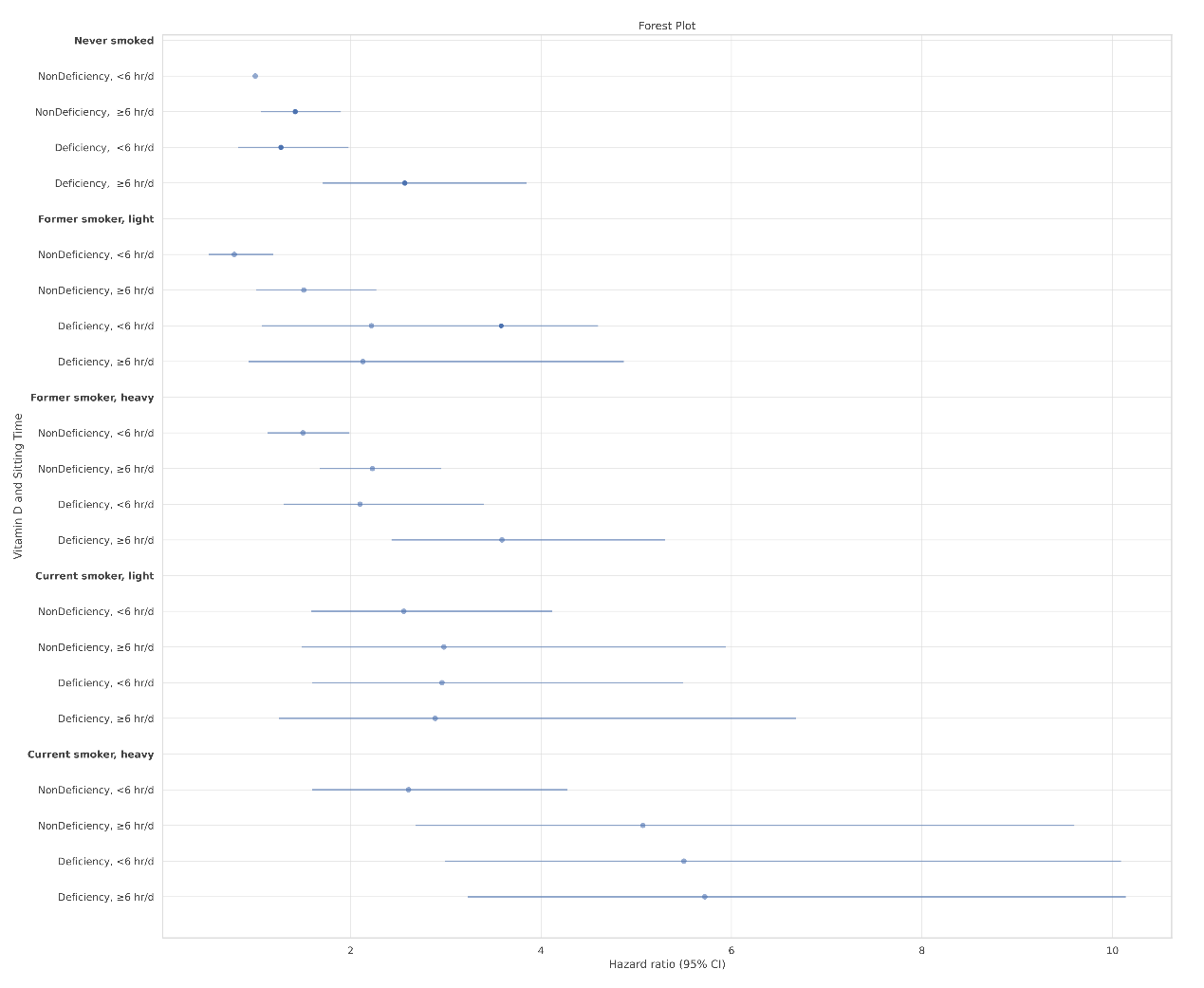


**Fig. S2.** Association of combined vitamin D status and sedentary time with all-cause mortality in smoking categories
